# Tetraspanin 6: a pivotal protein of the multiple vesicular body determining exosome release and lysosomal degradation of amyloid precursor protein fragments

**DOI:** 10.1186/s13024-017-0165-0

**Published:** 2017-03-10

**Authors:** Francesc X. Guix, Ragna Sannerud, Fedor Berditchevski, Amaia M. Arranz, Katrien Horré, An Snellinx, Amantha Thathiah, Takaomi Saido, Takashi Saito, Sundaresan Rajesh, Michael Overduin, Samir Kumar-Singh, Enrico Radaelli, Nikky Corthout, Julien Colombelli, Sébastien Tosi, Sebastian Munck, Isabel H. Salas, Wim Annaert, Bart De Strooper

**Affiliations:** 1 VIB Center for Brain and Disease research – VIB, Leuven, Belgium; 2 Center of Human Genetics and Leuven Institute for Neurodegenerative Diseases (LIND), KULeuven, Leuven, Gasthuisberg O&N, Belgium; 3 School of Cancer Sciences, University of Birmingham, Edgbaston, Birmingham, B15 2TT UK; 4 Department of Neurobiology, University of Pittsburgh Brain Institute, Pittsburgh Institute for Neurodegenerative Disease, University of Pittsburgh School of Medicine, Biomedical Science Tower 3, Room 6062, 3501 Fifth Avenue, Pittsburgh, PA 15213-3301 USA; 5 Laboratory for Proteolytic Neuroscience, RIKEN Brain Science Institute, Wako-shi, 351-0198 Saitama, Japan; 6 Department of Biochemistry, Faculty of Medicine & Dentistry, University of Alberta, Edmonton, Canada; 7 Molecular Pathology Group, Cell Biology & Histology, Faculty of Medicine, University of Antwerp, Antwerp, Belgium; 8 Institute for Research in Biomedicine (IRB Barcelona), The Barcelona Institute of Science and Technology, c. Baldiri Reixac 10, 08028 Barcelona, Spain; 9 Dementia Research Institute (DRI-UK), University College London, Queen Square, WC1N 3BG London, UK

**Keywords:** Alzheimer’s disease, Amyloid precursor protein, Intraluminal vesicles, Multivesicular bodies, Tetraspanin-6

## Abstract

**Background:**

The mechanisms behind Aβ-peptide accumulation in non-familial Alzheimer’s disease (AD) remain elusive. Proteins of the tetraspanin family modulate Aβ production by interacting to γ-secretase.

**Methods:**

We searched for tetraspanins with altered expression in AD brains. The function of the selected tetraspanin was studied in vitro and the physiological relevance of our findings was confirmed in vivo.

**Results:**

Tetraspanin-6 (TSPAN6) is increased in AD brains and overexpression in cells exerts paradoxical effects on Amyloid Precursor Protein (APP) metabolism, increasing APP-C-terminal fragments (APP-CTF) and Aβ levels at the same time. TSPAN6 affects autophagosome-lysosomal fusion slowing down the degradation of APP-CTF. TSPAN6 recruits also the cytosolic, exosome-forming adaptor syntenin which increases secretion of exosomes that contain APP-CTF.

**Conclusions:**

TSPAN6 is a key player in the bifurcation between lysosomal-dependent degradation and exosome mediated secretion of APP-CTF. This corroborates the central role of the autophagosomal/lysosomal pathway in APP metabolism and shows that TSPAN6 is a crucial player in APP-CTF turnover.

**Electronic supplementary material:**

The online version of this article (doi:10.1186/s13024-017-0165-0) contains supplementary material, which is available to authorized users.

## Background

Alzheimer’s disease (AD) is a devastating neurodegenerative disease and the first cause of human dementia [1]. Quantitative and qualitative alterations of the amyloid Aβ peptides together with tau-pathology, initiate a long cycle of cellular action and reaction eventually leading to severe neuronal loss [2]. Aβ peptides are generated from the transmembrane protein Amyloid Precursor Protein (APP) by proteolytic activities exerted by the β-secretase BACE1 and members of the γ-secretase family [3]. While mutations in APP and presenilins (the catalytic components of the γ-secretases complexes) alter Aβ production in familial AD cases [4], the mechanisms by which Aβ-peptide production, clearance and aggregation are altered in non-familial cases is of varied etiology [5–11].

Detergent resistant membrane (DRM) and tetraspanin-enriched microdomains (TEMs) play important roles in the regulation of the proteases that generate Aβ [12–17]. TEMs represent molecular assemblies of proteins and lipids based on multiple interactions involving four-transmembrane-domain proteins of the tetraspanin superfamily [18, 19]. In an unbiased proteomic analysis, our laboratory found that tetraspanins CD9 and CD81 (also known as TSPAN28 and TSPAN29) interact with components of the γ-secretase complex and modulate its activity towards APP [20]. Two other tetraspanins (TSPAN33 and TSPAN5) promote Notch activity [21]. Tetraspanins also play a role in the maturation and trafficking of ADAM10, one of the enzymes involved in the non-amyloidogenic cleavage of APP [22–24] and finally, interact with the γ-secretase complexes and ADAM10 or BACE1 to form supramolecular complexes in the membrane [17]. Beyond their involvement in the regulation of these proteolytic enzymes, tetraspanins play a crucial role in many other cellular processes, from the control of the endocytic trafficking to autophagic-induced cell death [18, 25–28]. Specifically, our interest in the role of tetraspanin-6 (TSPAN6) in sporadic AD was supported by observations from several independent groups which indicate that the mRNA levels of TSPAN6 are increased in the prefrontal cortex of AD patients and correlate with Braak stage progression [29–31]. Therefore, we decided to focus our studies on the role that TSPAN6 plays in the molecular pathogenesis of sporadic AD.

We demonstrate here that overexpression of TSPAN6 affects APP metabolism, but unexpectedly, this effect does not involve the γ-secretases. In contrast, we determined that TSPAN6 is enriched in multivesicular bodies (MVBs) and intraluminal vesicles (ILVs). Increasing TSPAN6 levels enhances endosomal size and leads to an increased number of intraluminal vesicles (ILVs) and increased secretion of exosomes containing the APP-CTF. TSPAN6 recruits the cytosolic adaptor syntenin through its PDZ1 domain which explains the increased exosome release, while the impaired degradation of proteins is due in part to the decreased fusion between autophagosomes and lysosomes caused by TSPAN6 expression. Thus the accumulation of APP-CTF is caused by a dual mechanism putting TSPAN6 in a pivotal point in the pathway of ILV towards exosomes or lysosomal degradation. The cellular pathology resembles the wide spread endolysosomal disturbances observed in neurons from patients suffering from sporadic AD.

## Methods

### Western blots

Total cell lysates were prepared in lysis buffer containing 50 mM HEPES pH 7.4, 150 mM NaCl, 1% TritonX100 (unless otherwise indicated) and complete protease inhibitors (Roche Applied Science). Post-nuclear fractions were taken, and protein concentrations were determined using standard BCA assay (Pierce Biotechnology). Proteins were separated on NuPAGE 4–12% Bis–Tris gels (Invitrogen) and transferred to nitrocellulose membranes for Western blot analysis. After blocking the membrane with 1% non-fat milk in T-TBS 1× (0.05 M Tris, pH 7.5; 0.15 M NaCl; 0.1% Tween20), the proteins were detected with the commercial antibodies: polyclonal anti-TSPAN6 antibody (1/500; Abgent), monoclonal MAB5232 anti-CTF-PS1 and monoclonal MAB1563 anti-NTF-PS1 (1/5000; Chemicon/Millipore), monoclonal 9C3 anti-Nct (1/5000), monoclonal 6E10 anti-Aβ (SIGNET), monoclonal anti-LC3β (D11) XP (1/1000; Cell Signalling), polyclonal anti-sAPPβ (1/1000; Covance), monoclonal anti-BACE1 D10E5 (1/1000; Cell Signalling), polyclonal anti-p62 (1/1000), monoclonal anti-FlagM2 antibody (1/4000; Sigma-Aldrich), monoclonal anti-βactin (1/5000; Sigma), polyclonal anti-APLP2 (1/1000; W2CT donated by Dominic Walsh), monoclonal anti-LRP1 EPR3724 (1/15000; Epitomics), monoclonal anti-Tsg101 (1/1000; Santa Cruz). The following in-house made antibodies: B63 for APP (1/10,000), B80 for Aph1a (1/1000) and B126 for Pen2 (1/2000).

Signals were detected using the chemiluminescence detection with Renaissance (PerkinElmer Life Sciences). Quantifications were performed by densitometry with the software AIDA

### Generation of the Tspan6 knockout mice and genotyping

For this KO line, ES cells derived from the 129/OlaHsd mouse substrain were used to generate chimeric mice. F1 mice were generated by breeding with C57BL/6 females. F2 homozygous mutant mice were produced by intercrossing F1 heterozygous males and females. The KO line has subsequently been backcrossed several times to C57BL/6.

In order to genotype the *Tspan6 KO* mice, tails were lyzed with KAPA Genotyping Kit (KAPA Biosystems) following the instructions of the company. For the PCR, 3 different primers were used: 5’- TGTGATCAAGGACTCAAGCTTGTAC-3’, 5’-CTTACTCACCAGTTTCAGCATCCAG-3’ and 5’-GGGTGGGATTAGATAAATGCCTGCTCT -3’.

### Immunohistochemistry on brain sections

Immunohistochemistry was performed as described in [32]. Briefly, antigen retrieval was performed in citrate buffer (0.018 M citric acid.H_2_O, 0.082 M sodium citrate, pH = 6) using microwave heating. Endogenous peroxidases and non-specific antigens were blocked by incubating sections in 0.3% H_2_O_2_ for 20 min followed by a 1:5 diluted normal horse serum block for 30 min. Sections were incubated overnight at 4 °C with primary antibodies: polyclonal anti-Tspan6 C-terminus (Abgent, 1:50 dilution), mouse monoclonal (mAb) anti-vGlut2 (Abcam, 1:2000 dilution) and mAb anti-GAD67 (Millipore, 1:500 dilution). Sections were further incubated with biotin conjugated secondary antibodies and extravidin conjugated HRP, each for 30 min at room temperature, and detected with 5, 5’ diaminobenzidine (Dako, Heverlee, Belgium). Images were captured using 40x objective and Olympus UC30 colour camera (Olympus, Antwerp, Belgium).

For double immunohistochemistry staining, combinations of anti-Tspan6 C-terminus antibody (Abgent, 1:50 dilution) with either anti-vGlut2 mouse monoclonal (Abcam, 1:2000 dilution) or anti-GAD67 mouse monoclonal (Millipore, 1:500 dilution) were incubated overnight and detected with DAG-Cy3 and DAM-Cy5 (Jackson Immunoresearch). Sections were counterstained with 5 μg/mL DAPI (Sigma-Aldrich, Diegem, Belgium) for 5 min and visualized with a dual spinning disk confocal microscope (Ultra*View* VoX, PerkinElmer, Seer green, UK) and images analysed using Volocity (PerkinElmer) essentially as described earlier [32].

### Mouse brain homogenates for western blot

Pieces of cerebral cortices of 1 year old *Tspan6* ^*+/Y*^ (*n* = 7) and *Tspan6* ^*-/Y*^ (*n* = 7) mice or 1 year old App^NL/F^ x *Tspan6 wt* (*n* = 5) and App^NL/F^ x *Tspan6 KO* mice (*n* = 6) were homogenized on ice in 0.5 M sucrose PKM buffer (100 mM Potassium phosphate; 5 mM MgCl_2_; 3 mM KCl; pH 6.5) containing 1% TritonX100 and 0.1% SDS. After removal of the debris by 30 minuts centrifugation at 14.000 rpm and 4 °C, 40 μg of total protein per sample was loaded on NuPAGE 4-12% Bis-Tris gels (Invitrogen).

### Mouse brain homogenates for ELISA

Pieces of mice cerebral cortices coming from 1 year old *Tspan6* ^*+/Y*^ (*n* = 7) and *Tspan6* ^*-/Y*^ (*n* = 7) mice were weighted and homogenized in 5 volumes of pre-filtered extraction buffer (0.4% diethanolamine, 50 mM NaCl, 1x Protease inhibitors (Roche)) with Fastprep (45 s at 6 m/s). The homogenates were centrifuged at 14.000 rpm for 5 min at 4 °C and the supernatants were ultracentrifuged at 90.000 rpm for 30 min at 4 °C. A 10% volume of neutralization buffer (0.5 M Tris-HCl, pH = 6.8) was added to the new supernatants before determination of the Aβ_40_ and Aβ_42_ content by ELISA. For normalization purposes, protein concentration was measured by standard BCA assay (Pierce Biotechnology).

### Primary neurons

Primary cultures of mouse cortical neurons were obtained from *Tspan6 KO* mice at E14.5. The procedure was carried out in accordance with the Ethic Committee of K. Leuven University (Ethische Commissie Dierproeven, KULeuven). Briefly, the cortical region of the brain was aseptically dissected and trypsinized for 15 min. Cells were seeded in phenol‐red MEM with L-glutamine (Invitrogen) plus 10% horse serum and 0.6% glucose into 0.1 mg/ml poly‐l‐lysine coated plates. After 120 min, medium was removed and neurobasal medium containing B27 supplement (NB-B27) was added.

### ELISA

For detection of human and mouse Aβ, an in-house ELISA sandwich was carried out. Briefly, 96-wells Nunc-Immuno plates (Nunc, Denmark) were coated overnight at 4 °C with JRF AB038 antibody for Aβ1‐38, JRF cAb040/28 antibody for Aβ40 or JRF Ab042/26 antibody for Aß42 (Janssen Pharmaceutica), all used at 1.5 mg/ml in PBS containing 0.1% casein (Casein Buffer). Plates were washed 5 times with Washing Buffer (PBS-0.05% Tween 20) before the addition of the samples or the standard curve made with consecutive dilutions (from 100 to 0.0003 ng/ml) of human or mouse Aβ40 and Aβ42 (rPeptide). Detection antibody was obtained from Janssen; huAB25‐HRPO. After overnight incubation at 4 °C and 5 time washes with the Washing Buffer, the samples were developed with a 0.02% TMB (tetramethylbenzidine) solution in Sodium Acetate (100 mM pH 4.9) containing 0.03% H_2_O_2_. The reaction was stopped with 0.2 N H_2_SO_4_ and read at 450 nm on a Perkin Elmer Envision 2103 multilabel reader.

### Immunoisolation of late compartments

Late compartments were isolated from HEK293 cells co-expressing an empty vector or myc-TSPAN6 together with LAMP1 fused to mRFP and to a double Flag-tag (LAMP1-mRFP-Flag) as previously described in Zoncu et al. [33] with small variations. Briefly, cells were harvested from 2 x T175 flasks per condition through scraping in cold PBS, spun down and resuspended in 1 ml of fractionation buffer: 50 mM KCl, 90 mM K-Gluconate, 1 mM EGTA, 5 mM MgCl_2_, 50 mM Sucrose, 20 mM HEPES, pH 7.4, supplemented with 2.5 mM ATP, 5 mM Glucose and protease inhibitors. Cells were mechanically broken by passing them through a 23G needle attached to a 1 ml syringe, then spun down at 2000 g for 10 min, yielding a post nuclear supernatant (PNS). The PNS was brought to 2 ml with fractionation buffer and subjected to immunoprecipitation with 50 μl of anti-FlagM2 affinity beads for 3 h at 4 °C. Late-compartments were in this way captured by the beads while the rest of organelles were washed out by 3 consecutive washes with fractionation buffer. Late-compartments bound to the beads were resuspended in loading buffer and proteins were separated on a 4-12% SDS-PAGE gel. After transferring proteins onto a nitrocellulose membrane by western blot, the enrichment of late compartments were analyzed with an anti-LAMP1 antibody, the content of APP and APP-CTF was evaluated with a polyclonal anti-APP antibody (B63) and the overexpression of TSPAN6 was determined by a polyclonal TSPAN6 antibody.

### Pulse-chase

HEK293 cells were seeded on 6-well plates and co-transfected with FlagAPP-C99 and an empty vector or TSPAN6. After 24 h cells were starved for 45 min and labeled with radioactive methionine/cysteine [35S] for 15 min. Cells were chased for indicated time points and lyzed in lysis buffer (1% TritonX100 in 100 mM NaCl, 50 mM HEPES pH 7.2 and protease inhibitors). FlagAPP-C99 was immunoprecipitated from the cell lysates using FlagM2-Sepharose beads (Sigma-Aldrich) and submitted to SDS-PAGE/western blot. Radiolabeled proteins were visualized with phosphoimaging.

### Proximity ligation assay (PLA)

PLA was carried out with the DuoLink In Situ Kit according to the manufacturer guidelines (Olink Bioscience). Briefly, HEK293 cells transfected with myc-TSPAN6 or left untransfected (technical control) were fixed in 4% paraformaldehyde on coverslips and blocked with blocking solution (0.3% TritonX100 and 5% goat serum in PBS) for 15 min at RT. A mouse monoclonal anti-myc antibody (9E10) and a rabbit monoclonal anti-APP antibody (Y188) were diluted 1/200 in PBS and applied to the samples for 2 h at RT in a humidity chamber. After removing the primary antibodies by 3 consecutive washes with PBS (5 min each), the samples were incubated for 1 h at 37 °C with the PLA Probes diluted 1/5 in PBS. After the incubation time, the unbound PLA probes were removed by consecutive washes with Buffer A (provided by the manufacturer), followed by the incubation of the samples for 30 min at 37 °C with the ligase. Buffer A was used to wash the samples before the addition of the polymerase-amplification solution. After 100 min incubation at 37 °C in a humidity chamber, the samples were washed with buffer B (provided by the manufacturer) and mounted for analysis by confocal microscopy.

### γ-Secretase assay with endogenous substrate

HEK293-APPwt cells were transfected with myc-TSPAN6, and 24 h post-transfection cell membranes were prepared. Cells were harvested and resuspended in hypotonic buffer (10 mM Tris, pH 7.3, 10 mM MgCl2, 1 mM EDTA and 1 mM EGTA) for 10 min on ice. Cells were then homogenized by passing 15 times through a 21G needle and centrifuged for 10 min at 100 g to pellet nuclei. The resulting supernatant was centrifuged 30 min at 16 000 g. Pelleted membranes were resuspended in citrate buffer (150 mM sodium citrate, adjust pH 6.4 with citric acid, complete protease inhibitors) and incubated at 4 °C (as negative control) or at 37 °C for 2 h. Analysis of γ-secretase products was performed with standard SDS-PAGE/western blot.

### γ-Secretase assay with exogenous substrate

HEK293 cells were transfected with myc-TSPAN6 for 24 h or treated with *siRNA-TSPAN6* for 48 h. Cell were homogenized in Buffer A (20 mM pipes pH7, 140 mM KCl, 0.25 M sucrose, 5 mM EGTA) with complete protease inhibitors (Roche Applied Science) and the microsomal membrane fraction was obtained by ultracentrifugation at 55.000 rpm at 4 °C. Cell-free assays were performed as described by Kakuda and colleagues [34] with some minor modifications. Briefly, microsomal membrane fractions solubilized in Buffer B (50 mM pipes, pH7, 0.25 M sucrose, 1 mM EGTA) containing 1% CHAPSO were mixed with recombinant APP-C99-3 × FLAG substrate (0.5 μM final concentration), 0.0125% phosphatidylethanolamine, 0.1% phosphatidylcholine, and 2.5% DMSO. Reactions were incubated at 37 °C for 4 h. Aβ species produced during the reaction were measured by ELISA and the levels of AICD produced were determined by SDS-PAGE electrophoresis and western blot with a mouse anti-FlagM2 and goat anti-mouse IR800 antibody. Infrared signals were detected using the Odyssey Infrared Imaging System.

### Exosomes isolation and quantification

HEK293 cells non-overexpressing (control) or overexpressing TSPAN6 were incubated for 2 days under growing conditions (at 37 °C and 5% CO_2_) with Exosomes-depleted medium obtained by overnight ultracentrifugation of 20% FBS-supplemented DMEM/F12 at 100.000 g and 4 °C and diluted 1:2 in FBS-free DMEM/F12. After 2 days the medium was collected and the death cells and other debris were removed by consecutive centrifugations at 4 °C at 200 g for 10 min, 2000 g for 10 min and 12000 g for 45 min. Finally the exosomes were obtained by ultracentrifugation of the supernatant at 110.000 g for 1 h at 4 °C. The pellet containing the exosomal fraction was washed once with pre-filtered PBS and posterior ultracentrifugation at 110.000 g for 1 h at 4 °C. The quality of the exosomal fraction obtained by this method was determined by comparing the protein expression of exosomal markers and non-exosomal proteins between total lysate and the exosomal fraction after electrophoretic separation of the samples on 4-12% SDS-PAGE and posterior western blot analysis with specific antibodies against the ER marker calnexin, Tsg101, TSPAN6 and actin. Determination of the number of exosomes present in the exosomal fraction was carried out by Nanosight.

### Electron microscopy

HEK cells and primary neurons grown on aclar coverslips were rinsed briefly and fixed in 1.3% glutaraldehyde in 66 mM cacodylate buffer for 2 h at RT before processing for electron microscopy. Then the cells were washed for 30 min in 0.1 M cacodylate buffer and post-fixed for 2 h at RT in 1% OsO_4_, 1.5% K_4_Fe(CN)_6_ in 0.1 M cacodylate buffer. Cells were rinsed again in 0.1 M cacodylate buffer and stained with 3% uranyl acetate for 1 h. Dehydration was performed at RT by transferring the cells to 35%, 50%, 70%, 90% and two steps of 100% ethanol for 10 min each. The cells were then embedded in EMbed812 and resin blocks were sectioned on an ultramicrotome (Leica Ultracut UCT, Leica Mycrosystems). Ultrathin sections (70 nm) were mounted on copper grids and imaged using a JEM-1400 transmission electron microscope (JEOL), equipped with an 11Mpixel Olympus SIS Quemesa camera. Quantifications of the area and number of subcellular compartments were performed with the image processing software ImageJ (Fiji distribution).

### Lentiviral vectors production and transduction of neurons in culture

The vectors psPAX2 and pMD2.G were constructed and provided by D. Trono (Geneva, Switzerland); the vector pLVx-puro was used to subclone *Tspan6*.

Lentiviral particles were produced by using TransIT-LT1 transfection protocol on 60% confluent HEK293. Cells were transfected with a plasmid mix containing the vector pLVx encoding Tspan6 (LV-Tspan6) or empty pLVx (LV-cnt), the packaging construct psPAX2 and the envelope plasmid pMD2G-VSVG (at the following ratio 1.5:2:1). After 4 h post-transfection, the medium was replaced by fresh growth medium and cells were left for 24 h to produce viral particles. The 24 h-medium was harvested and fresh growth medium was added to the cells for additional viral production. After another 24 h the medium was harvested and mixed with the first 24 h medium. The medium was ultracentrifugated at 50.000 g for 2 h at 16 °C to pellet down the viral particles. Finally, the lentiviral vesicles were resuspended in sterile 100 μL PBS.

Primary cortical neurons derived from *Tspan6* ^*+/Y*^ and *Tspan6* ^*-/Y*^ E14.5 embryos were plated in 12-wells plates and transduced at DIV 6 by replacing the growth medium (NB-B27) by fresh neurobasal medium without B27 (NB) containing 3 μL of LV-TSPAN6 or LV-cnt. After 4 h the medium was replaced by the original NB-B27 and the neurons were left for 2 days to express the gene of interest. At DIV 9 the medium was replaced by experimental medium (NB containing only glutamate) and 24 h later it was collected for Aβ determination and neurons were lyzed in lysis buffer for western blot analysis or fixed in 1.3% glutaraldehyde in 66 mM cacodylate buffer for EM analysis.

### Confocal laser scanning microscopy and quantification

Cells were plated on glass coverslips, transfected 24 h and processed for indirect immunolabeling the next day as in [35]. Images were captured on a confocal microscope (Leica TCS SP5 II, Leica Microsystems) connected to an upright microscope, using an oil-immersion plan Apo 60x A/1.40 NA objective lens. Image acquisition was performed with LAS (Leica Microsystems Gmbh, Wetzlar, Gemany) and further processed with ImageJ (Rasband, W.S., ImageJ, U. S. National Institutes of Health, Bethesda, Maryland, USA, http://imagej.nih.gov/ij/, 1997–2016). The ImageJ plugin JACoP (Fabrice P. Cordelieres, Institut Curie, Orsay, France. Susanne Bolte, IFR 83, Paris, France) was used for signal colocalization by overlap quantification (Manders coefficients). Images were acquired with identical settings and regions of interest covering whole cells were used for quantification [36]. Data are expressed as mean ± s.e.m. from n cells

### Study of autophagic flux

HEK293 cells were seeded on 12-well plates and transfected with an empty vector or TSPAN6. After 24 h the cells were washed once with *Earle's Balanced Salt Solution* (EBSS) medium and incubated with EBSS medium for the time indicated with or without 10 μM Bafilomycine A1. After the incubation time, the cells were lyzed in lysis buffer at 4 °C and proteins were analyzed by SDS-PAGE electrophoresis and western blot. The activation of autophagy was analyzed with a polyclonal antibody anti-LC3β (Cell Signalling) and a polyclonal antibody against p62. The effect of the activation of autophagy on the degradation of APP-CTF or PS1-CTF (negative control) was evaluated with a polyclonal antibody against APP (B63) and a monoclonal antibody against PS1-CTF (Millipore). The overexpression of TSPAN6 was evaluated with a polyclonal antibody against TSPAN6 (Abgent).

### Cell-based assay for Notch

HEK293 cells were seeded on 6-wells plates (800.000 cells/well). For the evaluation of the effect of TSPAN6 overexpression on the γ-secretase-mediated cleavage of Notch, cells were co-transfected with NotchΔE and an empty vector or TSPAN6. For the study of the effect of TSPAN6 downregulation on Notch processing, cells were co-transfected with siRNA against TSPAN6 (*siRNA-TSPAN6*) or a scrambled siRNA (siRNA-cnt) and NotchΔE. The cells were treated with 10 μM lactacystine overnight to allow the accumulation of the NICD product before lyzing the cells in lysis buffer for 20 min. After lysis, the samples were spun down for 14.000 rpm for 15 min at 4 °C and 30 μg of total protein of the supernatant was run on a 12% MES gel and transferred to a nitrocellulose membrane by western blot. Analysis of NotchΔE cleavage was evaluated with a monoclonal antibody against cleaved Notch1 (Val1744; 1/1000) and a monoclonal antibody against myc (9E10; 1/15000). Goat anti-mouse IR700 antibody and goat anti-rabbit IR800 antibody were used to analyze the infrared signals of the bands with the Odyssey Infrared Imaging System.

### Real‐time PCR

Total RNA was extracted from HEK293 cells expressing an empty vector or TSPAN6 with the miRVana kit (Thermo Fisher Scientific). Briefly, cells were collected in 1 ml ice-cold Trizol after a brief wash in ice-cold PBS and homogenized with a 23G syringe needle on ice. After the addition of 200 μL chloroform the homogenate was centrifuged at 12,000 g for 1 min at RT and the aqueous phase (~400 μL) was transferred to a new tube and mixed with 1.25 volumes of ethanol 100% at RT. The mixture was loaded onto a column and spun at 12,000xg for 40 s at RT. After several washes of the column with washing solutions, total RNA was eluted with RNA-free water (50 ng/μL). To obtain the cDNA, the reverse transcription mix contained 4 μl of total RNA (200 ng), 4 μl of 5× reaction buffer, 2 μl of enzyme mix and 10 μl of water. The reactions were incubated at 42 °C for 1 h followed by inactivation at 95 °C for 5 min.

For real-time PCR determination of the APP and BACE1 mRNA levels, samples were diluted 40x and 5 μl product was assessed in a PCR reaction mix containing 10 μl of SYBR Green master mix. Real‐time PCR was performed on 96‐wells plates using the LightCycler 480 Real‐Time PCR system (Roche, Indianapolis, IN, USA). The PCR conditions were 95 °C, 10 min followed by 45 cycles at 95 °C, 10 s and 55 °C, 20 s. The crossing point (Cp) was determined by using the second derivative method and data were normalized with actin. Fold changes and statistical significance were calculated using one‐way ANOVA.

#### NMR spectroscopy and C-terminal peptide ligand titration

For the heteronuclear magnetic resonance spectroscopy (NMR) analysis of syntenin-1 and TSPAN6 interaction, uniformly 15 N-labeled syntenin-1 PDZ tandem domain (113 to 273), referred to as “syntenin-1 PDZ12,” was expressed as a GST fusion in Escherichia coli BL21 (DE3) at 25 °C in M9 minimal medium using 15NH4Cl as the sole nitrogen source. The purification of GST-fused protein was performed as reported previously [37]. The NMR samples contained 200 μM protein, 150 mM NaCl, 500 μM TCEP [Tris(2-carboxyethyl)phosphine], and 50 μM AEBSF in 50 mM Tris buffer (pH 7.5). All NMR spectra were recorded at 30 °C on a Varian Inova 800-MHz spectrometer equipped with a room temperature 5-mm 1H/13C/15 N z-axis pulse field gradient probe, with data processed using the Azara package and analyzed using Ansig [38]. Synthesized C-terminal TSPAN6 peptide was purchased from Sigma-Genosys and contained an extra Arg residue at the N terminus, to improve peptide solubility. Titrations of syntenin-1 PDZ12 proteins with C-terminal TSPAN6 peptide were conducted by recording a series of 1H,15N HSQC spectra of 15 N-labeled protein (200 μM), with increasing molar concentrations of the peptide ligand, up to a protein-to-peptide ratio of 1:8. The combined backbone 1H and 15 N chemical shift changes, Δδ, were calculated as reported earlier [39].

### Syntenin contructs

Constructs expressing syntenin wt and mutants were obtained as described previously [40].

### Brain clearing and 3D Lightsheet imaging

The App^NL-F/NL-F^ mice were generated before [41]. Fixed brains from 1 year old App^NL/F^ x *Tspan6 KO* and App^NL/F^ x *Tspan6 Wt* mice were permeabilized with 1% Triton X-100 at room temperature for 24 h. Amyloid plaques were stained in 0.1% Thioflavin-S solution for 24 h. Then we rinsed them 2 times in PBS and dehydrated the tissue in an ethanol series (30%, 50%, 70%, 80%, 96% and 2 times in 100%, 1 day incubation time each) at room temperature. We rinsed the brains in 100% hexane for 1 h and then transferred them into a clearing solution of 1 part Benzyl Alcohol (Sigma) in 2 parts Benzyl Benzoate (Sigma), aka BABB. We stored whole brains in the clearing solution for at least 2 days at room temperature before imaging.

After clearing of intact mouse brains, imaging was achieved using a custom made Lightsheet microscope. The detection unit is equipped with a Nikon Macroscope AZ100M (Nikon Europe, The Netherlands), an ORCA R2 camera (Hamamatsu, Japan) and single bandpass and longpass filters (SemRock, Rochester, NY). The magnification used to resolve amyloid plaques was in a range of 9x-12x and cortical volumes of about Illumination was achieved with a 50 mm focal length cylindrical lens providing a lightsheet thickness in the order of 5–10 microns, with a slitted and collimated beam of 488 nm and 561 nm solid state lasers.

### Image analysis of 3D volume

A custom ImageJ macro was used to extract, count and measure the individual volumes of the amyloid plaques in the acquired image Z-stacks. The macro performs the detection of candidate plaques (hits) by extracting 3D objects around significant intensity maxima. It then displays local volumes (boxes) cropped around hit locations, both in Thiaflovin and control (autofluorescence) channels, into an image montage. The user can navigate to hit locations within the full volume by interacting with the montage and discards invalid hits based on visual inspection. Intensity Z-projections of the cropped volumes is also displayed to quickly guide the user to suspicious hits. Validated amyloid plaques are finally automatically counted and their individual volumes exported to coma separated text files for further statistical analysis.

## Results

### TSPAN6 alters cellular proteostasis

**Fig. 1:**
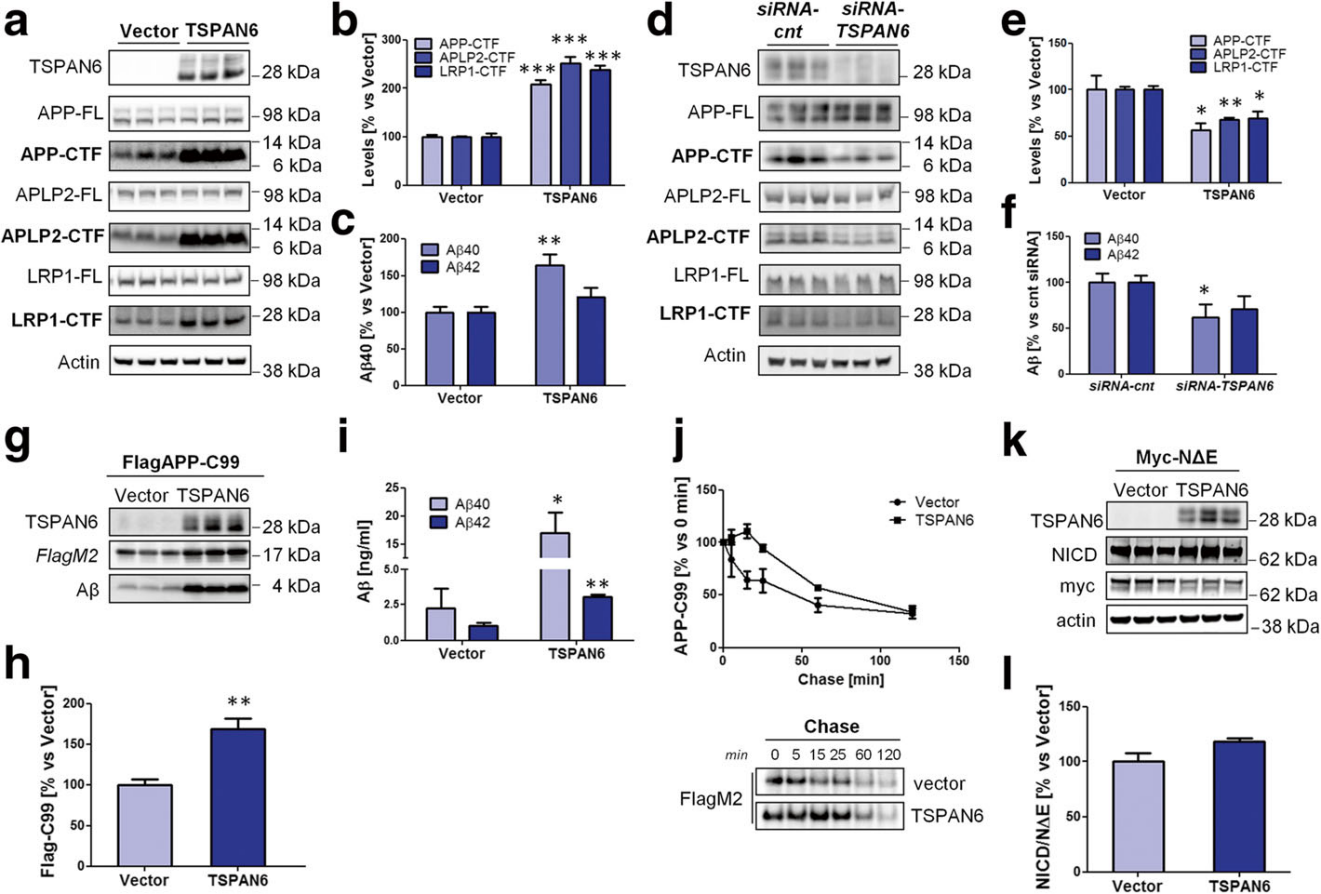
TSPAN6 affects the normal proteostasis of the cell. **a** TSPAN6 was transfected in HEK-APPwt (*n* = 3 technical repeats) and analyzed by western blot using antibodies against APP, APLP2 and LRP1. The CTF levels of APP (APP-CTF), APLP2 (APLP2-CTF) and LRP1 (LRP1-CTF) were elevated by overexpressing TSPAN6. No changes in the levels of full-length proteins for APP (APP-FL), APLP2 (APLP2-FL) and LRP1 (LRP1-FL) were observed. **b** Quantification of bands in panel **a** confirmed the increase in CTF levels of APP, APLP2 and LRP1. **c** Aβ determination by ELISA in the 24 h conditioned medium of HEK-APPwt cells like those in panel **a** shows increased Aβ_40_ levels (*n* = 3 experiments). **d** Cell lysates of HEK-APPwt cells treated 48 h with a scrambled siRNA (siRNA-cnt) or a siRNA against *TSPAN6* (*siRNA-TSPAN6*) were analyzed by western blot as in panel **a** (*n* = 3 experiments). In contrast to panel **a**, the levels of APP-CTF, APLP2-CTF and LRP1-CTF were strongly reduced in siRNA-*TSPAN6*-treated cells. **e** Quantification of bands in panel **d. f** Aβ levels determined by ELISA in the 24 h-conditioned medium of the cells in panel **d** show a significant decrease in Aβ_40_ levels. **g** HEK293 cells were co-transfected with FlagAPP-C99 and TSPAN6 or an empty vector and lysates analyzed by western blot with antibodies against FlagM2 and 6E10 (*n* = 3 experiments). **h** Quantification of FlagAPP-C99 from panel **g. i** Aβ in the 24 h conditioned medium of the cells from panel **g** measured by ELISA. TSPAN6 overexpression increases Aβ_40_ and Aβ_42_ levels. **j** Transfected HEK293 analyzed by pulse-chase labeling. Cells were incubated for two hours with ^35^S-Met/Cys and FlagAPP-C99 was immunoprecipitated at specific time-points with FlagM2-affinity gel. The density of the bands in the autoradiography was quantified and plotted (*n* = 3 experiments). This shows a slower disappearance of FlagAPP-C99 during the first 30 min in cells overexpressing TSPAN6. **k** HEK293 cells co-transfected with myc-tagged NotchΔE and TSPAN6 or an empty vector (*n* = 3 experiments). TSPAN6 overexpression do not change myc-NΔE levels. **l** Quantification of panel **k** shows no differences in terms of NICD production in TSPAN6. Statistical significance was determined by *t*-test (*p <0.05, **p <0.01, ***p <0.001)

Given that several studies [29–31] show that *TSPAN6* transcripts increase in late Braak stages of the Alzheimer’s disease brain, we first overexpressed a myc-tagged form of TSPAN6 (myc-TSPAN6) in human embryonic kidney (HEK293) cells. As we anticipated that tetraspanins affect γ-secretase activities, we selected APP and two other known substrates of γ-secretases, i.e. APLP2 and LRP1 for further analysis. Levels of APP, APLP2 and LRP1 full length proteins were not affected upon TSPAN6 overexpression in HEK cells stably expressing APP (HEK-APPwt) (Fig. 1a). However a strong accumulation of their respective CTFs (APP-CTF, APLP2-CTF and LRP1-CTF; Fig. 1a,b) was seen. Aβ_40_ but not Aβ_42_ was increased in the conditioned medium of TSPAN6 transfected cells (Fig. 1c), suggesting a preserved γ-secretase activity. The increased levels of substrate (APP-CTF) might explain the increased Aβ_40_ but some effects on the processivity of the enzyme cannot be excluded given the selective effect on Aβ_40_. γ-Secretase expression was not affected by TSPAN6 overexpression Additional file 1: Figure S1a) and no effects were seen in vitro in γ-secretase activity assays using microsomal fractions of control, TSPAN6-overexpressing or TSPAN6-downregulated HEK-APPwt cells (Additional file 2: Figure S2a,b). Interestingly, the expression levels of BACE1 and the consequent generation of sAPPβ in early endosomes (EE) [35] were elevated by TSPAN6 while no changes in the levels of ADAM10 and sAPPα were found (Additional file 1: Figure S1a-c). In contrast, downregulation of endogenous TSPAN6 in HEK-APPwt cells using siRNA gave the opposite results: reduced CTF levels of APP, APLP2, LRP1 (Fig. 1d,e) and reduced BACE1- and sAPPβ-levels (Additional file 1: Figure S1d-f), with no change in the levels of the γ-secretase components.

To gain insight into the cellular mechanism of the altered proteolysis we focused on the accumulation of the APP-CTFs which are generated by α- or β-secretases (APP-C83 and APP-C99, respectively). We co-transfected HEK293 cells with similar amounts of Flag-tagged APP-C99 (FlagAPP-C99) and an empty vector (negative control) or TSPAN6 (Fig. 1 g). Both FlagAPP-C99 and secreted Aβ levels were enhanced by TSPAN6 (Fig. 1 g-i), consistent with the altered proteolysis of the APP-CTF fragments. We also tested the effect of TSPAN6 on myc-tagged Notch-ΔE, a synthetic fragment from Notch that is used as a direct substrate for γ-secretase, and which can be compared to APP-CTF. The Notch-CTF did not change and generation of the Notch intracellular domain (NICD) by γ-secretase was not affected following overexpression or down regulation of TSPAN6 (Fig. 1 k,l; Additional file 2: Figure S2c,d), indicating specificity of the TSPAN6-mediated effect on APP-CTF, APLP1-CTF and LRP1-CTF relative to the Notch-CTF.

The turn-over of FlagAPP-C99 was further investigated with pulse-chase experiments. Cells were radiolabeled with ^35^S-Met/Cys. At the time points indicated, FlagAPP-C99 was immunoprecipitated and analyzed by SDS-PAGE and autoradiography. TSPAN6-overexpression resulted in a delay in the degradation of FlagAPP-C99 which was overcome after 30 min (Fig. 1j). The endogenous protein can accumulate in presence of high TSPAN6 levels assuming that APP-C99 is continuously being produced and considering the delayed start of its degradation. Altogether these data strongly suggest that TSPAN6 affects specifically the degradation of some proteins, including APP-CTF, APLP2-CTF and LRP-CTF.

### APP-CTFs accumulate in endosomal compartments in TSPAN6-overexpressing cells

**Fig. 2:**
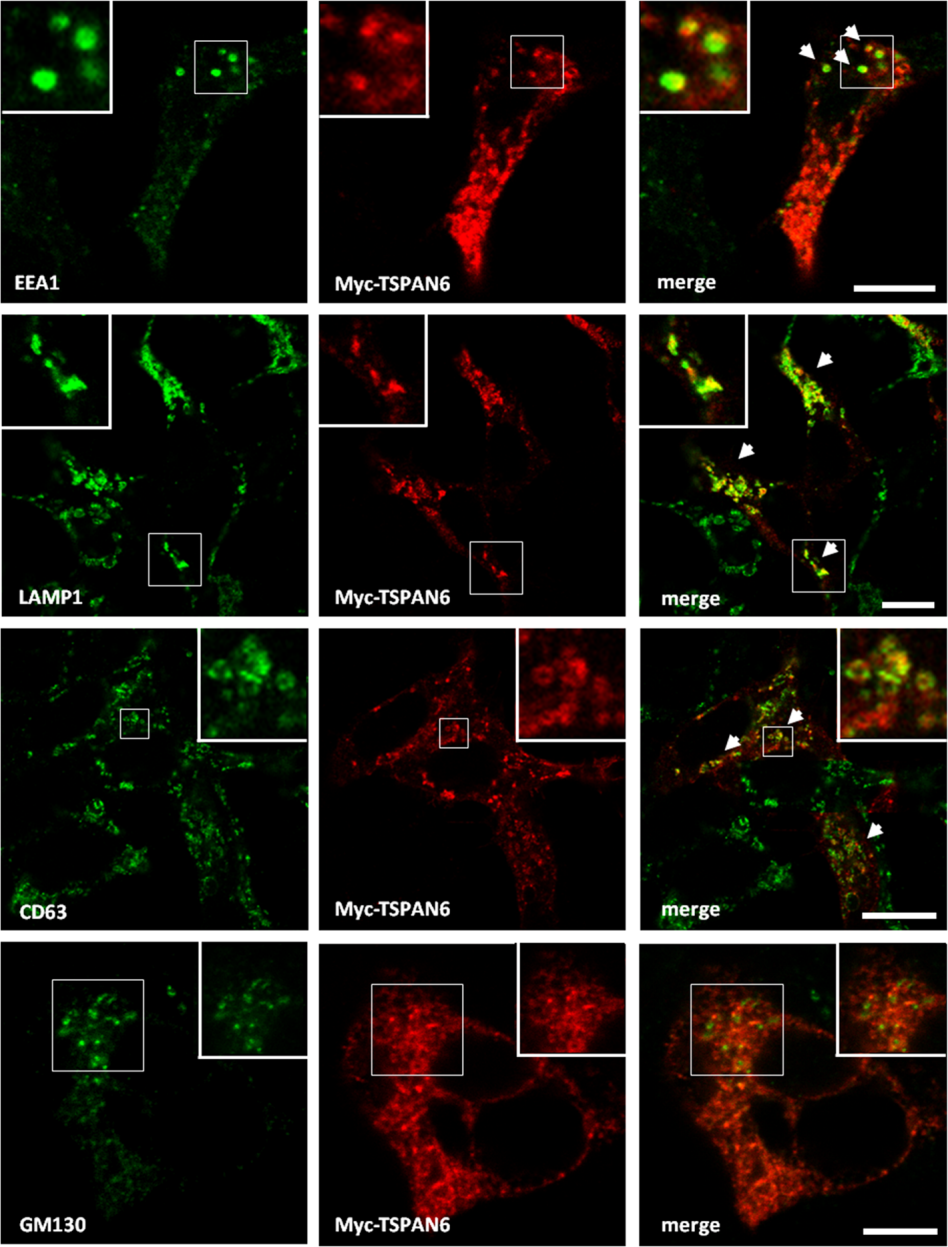
Subcellular localization of TSPAN6 in HEK293 cells. HEK293 cells were transfected with myc-tagged TSPAN6 (myc-TSPAN6), fixed and immunostained for EEA1 as an early-endosomes marker (upper panels), LAMP1 and CD63 as markers for late-compartments (middle panels), and GM130 to visualize the Golgi organelle (lower panels). Myc-TSPAN6 (detected with a rat monoclonal anti-myc antibody) shows a high degree of co-localization (arrows) with late-compartments (detected with a mouse monoclonal antibody against LAMP1 and a rabbit polyclonal antibody against CD63), and a lower co-localization with early endosomes (detected with a mouse monoclonal antibody against EEA1). Low co-localization was observed with the Golgi organelle (detected with a monoclonal antibody against GM130). Scale bars = 10 μm

**Fig. 3:**
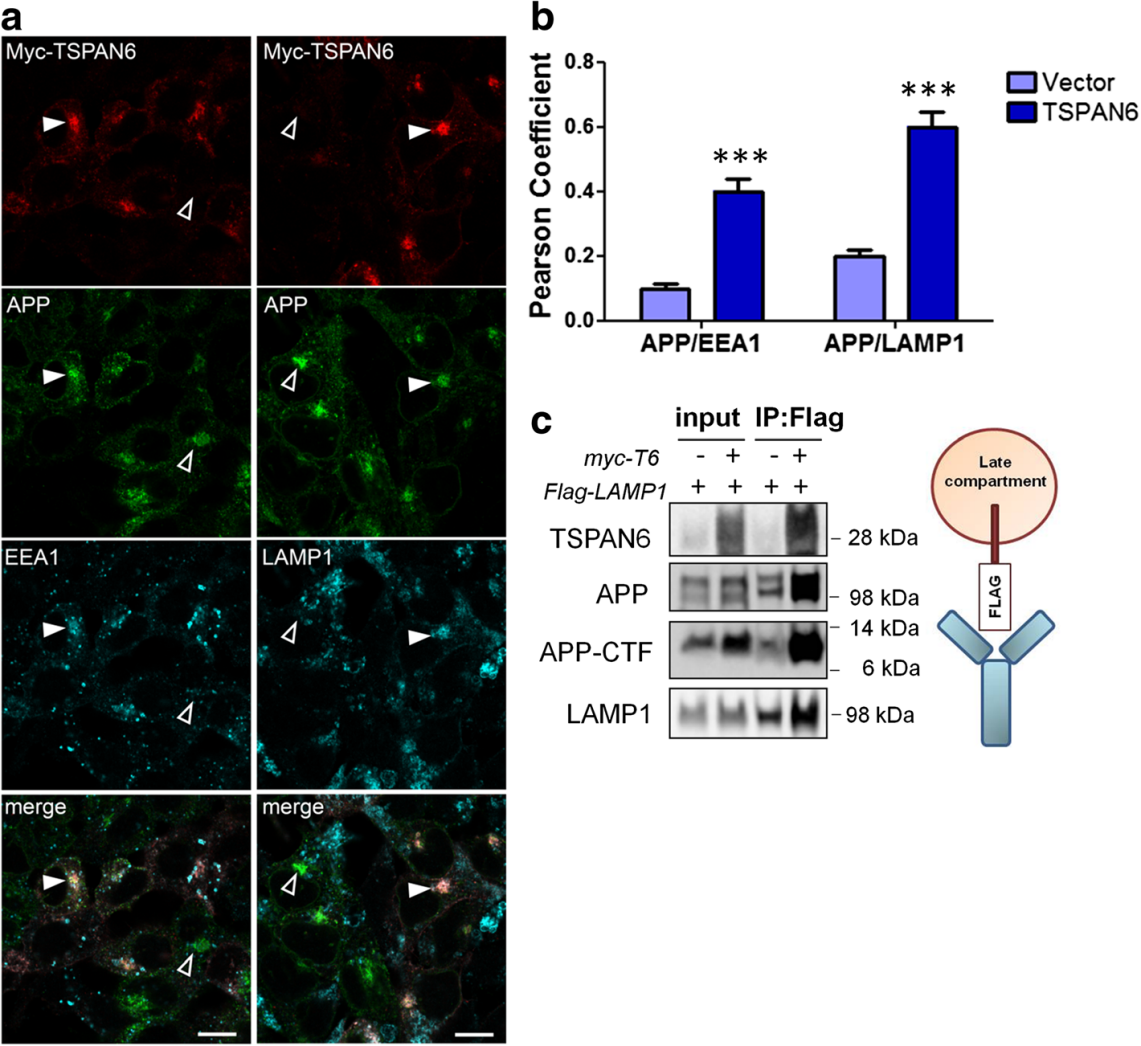
APP-CTF accumulates in endosomal compartments in TSPAN6-overexpressing cells. **a** Immunofluorescence microscopy of HEK293 cells overexpressing myc-tagged TSPAN6, fixed and probed with a rat monoclonal antibody against myc, a rabbit monoclonal antibody against APP-CTF (Y188), and mouse monoclonal antibodies against EEA1 and LAMP1 to visualize early and late endosomal compartments, respectively. Cells overexpressing myc-TSPAN6 show more APP co-localizing with EEA1 and LAMP1 (filled arrowheads) compared to non-overexpressing cells (empty arrowheads). **b** Quantification of the number of cells non-overexpressing or overexpressing TSPAN6 showing co-localization of APP-CTF with early (EEA1) or late endosomal (LAMP1) compartments. **c** Immunoisolation of LAMP1-containing compartments as described [33], after overexpressing Flag-tagged LAMP1 in non-overexpressing or overexpressing myc-tagged TSPAN6. The total inputs and the immunoisolated compartments were analyzed by western blot with specific antibodies against APP, APP-CTF, TSPAN6 and LAMP1. Statistical significance was determined by *t*-test (***p <0.001)

We next investigated the subcellular compartmentalization and accumulation of APP-CTF upon TSPAN6 overexpression. As shown in Fig. 2, TSPAN6 is mainly present in LAMP1 positive organelles and to a lesser extent in EEA1 positive organelles. By contrast, we found no-colocalization with the Golgi marker GM130. APP-CTFs show strong co-localization with TSPAN6 in LAMP1 cellular compartments (Fig. 3a,b). This co-localization was specific for the CTFs of APP since N-terminal epitopes of APP show only a weak co-localization with myc-TSPAN6 (Additional file 3: Figure S3). Remarkably, overexpression of myc-TSPAN6 increases the co-localization of endogenously expressed APP-CTF with LAMP1 by threefold and with EEA1 by twofold (Fig. 3a,b). We confirmed the enrichment of APP-CTFs in LAMP1 positive organelles by immunoisolation of these compartments after expression of Flag-tagged LAMP1 (Flag-LAMP1) and using Flag-tag antibody coupled to beads [33]. APP-CTF was found to enrich in the immunoprecipitated LAMP1 positive organelles when cells overexpressed TSPAN6 (Fig. 3c), confirming that TSPAN6 increases the levels of APP-CTFs in these compartments.

### TSPAN6 favors the generation of exosomes

**Fig. 4:**
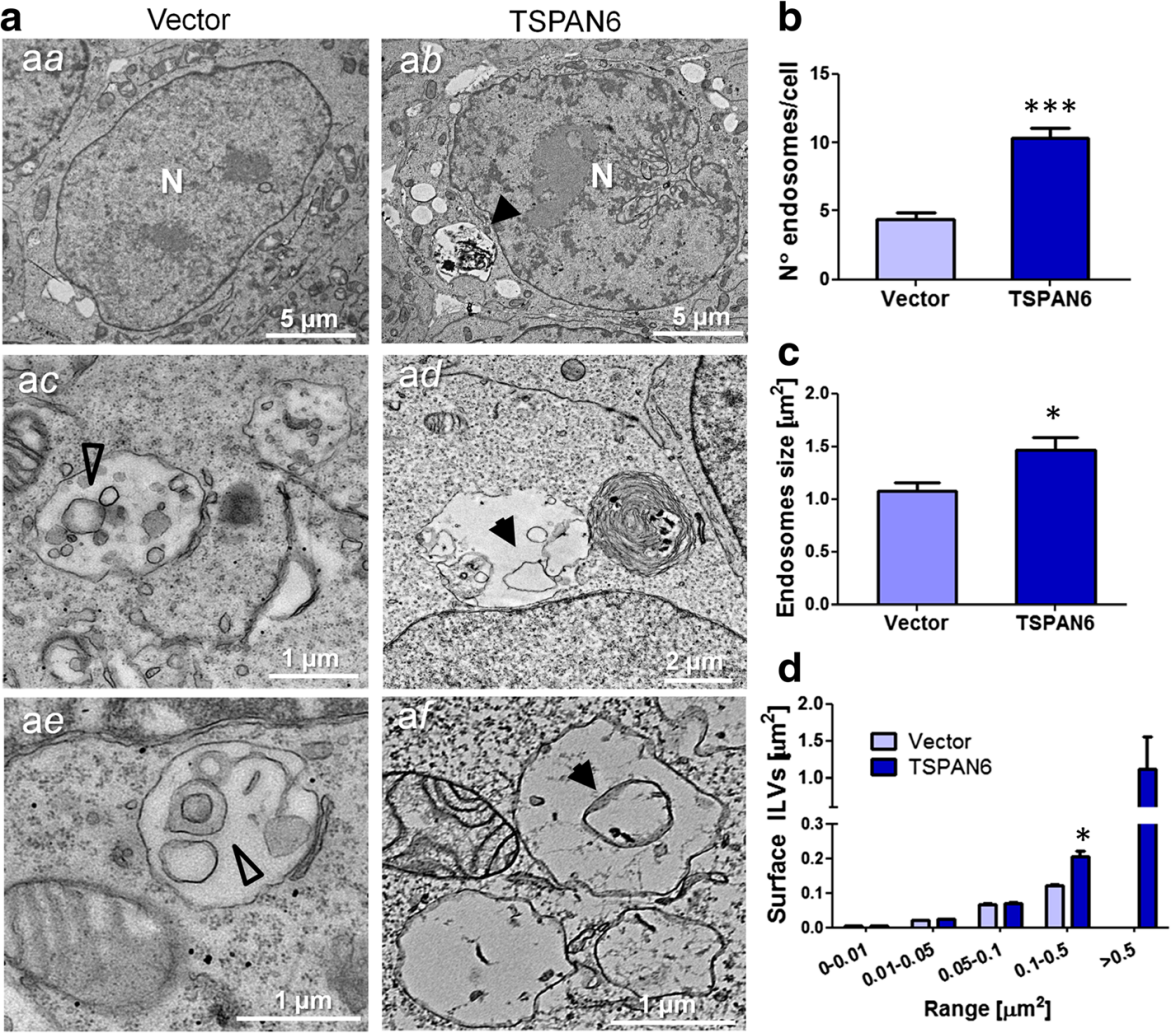
Endosomal alterations in TSPAN6-overexpressing cells. **a** EM images showing enlarged compartments in HEK293 cells overexpressing TSPAN6. HEK293 cells expressing an empty vector (control) present endosomes (**a** *a*) showing a normal aspect and multivesicular bodies (MVBs; **a** *c* and **a** *e*) containing normal-sized intraluminal vesicles (ILVs, empty arrowheads). In comparison, HEK293 cells overexpressing TSPAN6 show a higher number of endosomal compartments (**a** *b*), some of them with non-degraded material in the lumen (**a** *b,* arrowheads) and MVBs with enlarged ILVs (**a**d and **a** *f*; arrowheads). The nucleus is indicated with an N. **b** Quantification of the number of endosomes from TEM images of HEK293 cells like those shown in panel **a**. Overexpression of TSPAN6 increases the number of endosomal compartments per cell (*n* = 72 control cells; *n* = 59 TSPAN6-transfected cells; 2 independent experiments). **c** Analysis of the size of endosomal compartments from TEM images. Larger endosomal compartments are found in HEK293 cells overexpressing TSPAN6 in comparison to controls cells expressing an empty vector (*n* = 167 endosomes for control cells; *n* = 267 for TSPAN6-transfected cells; 2 independent experiments). **d** Determination of the surface of ILVs from TEM images like those shown in panel **a**. All the ILVs from the control or the TSPAN6-overexpressing cells were grouped according to their size in μm^2^ (*n* = 202 ILVs from control cells; *n* = 204 ILVs from TSPAN6-overexpressing cells; 2 independent experiments). This demonstrates enrichment for larger structures in the TSPAN6 overexpressing cells. The mean of the surface plotted for each range shows larger ILVs in cells overexpressing TSPAN6 when comparing ILVs larger than 0.1 μm^2^. Statistical significance was determined by t- test (*p <0.05, ***p <0.001)

These studies indicate that TSPAN6 impairs degradation of the APP-CTF that accumulates in endosomal compartments. Thus we decided to study these organelles at the ultrastructural level on HEK293 cells overexpressing TSPAN6 by Transmission electron microscopy (EM). EM analysis revealed that expression of TSPAN6 led to a duplication of the number of endosomal structures. These were moreover 40% larger than those observed in control cells (Fig. 4a-c). Furthermore, in TSPAN6-expressing cells intraluminal vesicles (ILVs) of multivesicular bodies (MVBs) were somewhat larger when compared to control cells (Fig. 4 a*c*-a*f*). These data were further confirmed by stereological analysis that demonstrated that the ILVs present in cells overexpressing TSPAN6 have a larger surface compared to the largest ILVs found in control cells and also appear to display more empty space in EM (Fig. 4d).

**Fig. 5:**
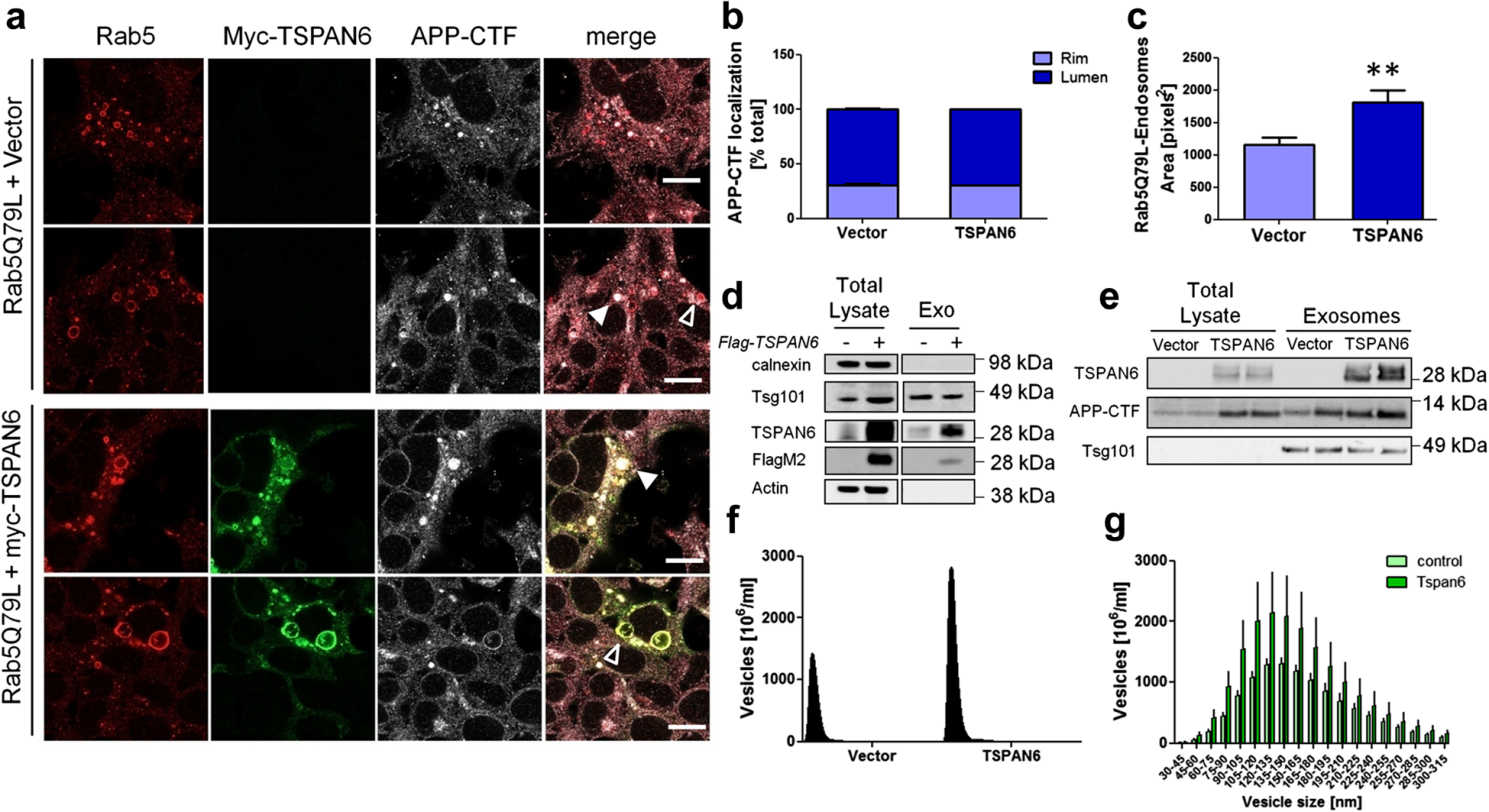
TSPAN6 is found in ILVs and enhances the formation of exosomes. **a** Representative staining of 2 independent experiments showing HEK293 cells overexpressing the Rab5^Q79L^ mutant together with an empty vector (upper panels) or myc-TSPAN6 (lower panels). Immunofluorescence studies were carried out with monoclonal antibodies against myc (to detect TSPAN6), APP-CTF and Rab5. APP-CTF is observed in the lumen (filled arrowheads) and the limiting membrane (empty arrowheads) of endosomes in both conditions; however, cells overexpressing myc-TSPAN6 show enlarged endosomes. Scale bar = 10 μm. **b** Percentage of APP-CTF in the lumen or the rim of endosomes in cells overexpressing an empty vector (*n* = 109 endosomes; 2 independent experiments) or myc-TSPAN6 (*n* = 106 endosomes; 2 independent experiments). **c** The average area (pixels^2^) of Rab5^Q79L^ endosomes in cells overexpressing myc-TSPAN6 (*n* = 75 endosomes; 2 independent experiments) was increased in comparison to control cells expressing an empty vector (*n* = 65 endosomes; 2 independent experiments). **d** Western blot analysis of total HEK293 lysates (transfected with Flag-TSPAN6 or left untransfected) or the exosomal fraction (Exo) isolated from their conditioned medium. The blot was probed with a monoclonal antibody against FlagM2 and a polyclonal antibody against TSPAN6 to assess the overexpression of protein, a monoclonal antibody against calnexin (non-exosomal protein), a polyclonal antibody against Tsg101 (exosomal protein) and a monoclonal antibody against actin. **e** The western blot of total HEK293 lysates and their exosomal fractions shows no differences in the APP-CTF levels for the same amount (2.5 μg) of exosome lysates when TSPAN6 is overexpressed. **f** Quantification of the concentration of secreted exosomes (vesicles/ml) by the nanovesicles tracking system Nanosight from control HEK293 cells or overexpressing TSPAN6 (3 independent experiments per condition), shows that TSPAN6 increases the secretion of exosomes. **g** Graph showing the distribution of the concentrations of the vesicles grouped by size (nm) and found in the exosomal fractions of TSPAN6-overexpressing HEK293 cells and control cells (data from panel **f**). TSPAN6-overexpressing cells show a higher concentration for all the vesicle sizes. Statistical significance was determined by *t*-test (**p <0.01)

APP-CTF degradation can occur via sorting into ILVs from the rim of the MVB followed by fusion to lysosomal or autolysosomal compartments. Given the altered ILVs morphology found by EM analysis, we explored a possible defect in this pathway as a TSPAN6-induced mechanism leading to APP-CTF accumulation. We expressed the constitutively active GTP-locked mutant form of the early endosomal marker Rab5 (Rab5^Q79L^). Rab5^Q79L^ blocks recycling from EEs resulting in enlarged EEs through fusion events without affecting the formation of ILVs [42]. As shown in Fig. 5, TSPAN6 co-localizes with Rab5^Q79L^ at the rim of such enlarged endosomes but is also found in the lumen (Fig. 5a). Interestingly APP-CTF distribution between the rim and the lumen of MVBs was not different between TSPAN6-overexpressing and control cells (Fig. 5a,b), indicating that TSPAN6 does not interfere with the sorting of APP-CTF into ILVs (Fig. 5b). The enlarging effect of Rab5^Q79L^ on endosomes is however significantly enhanced in the presence of TSPAN6 (Fig. 5c).

Ubiquitinated proteins are recognized by ESCRT-proteins and sorted to ILVs in MVB. Further degradation occurs when the latter fuse to autophagosomes and lysosomes. Alternatively cargo sorted to tetraspanin-enriched microdomains in the limiting membrane of the endosome may go to ILVs destined for exosome release in an ESCRT-independent manner [43]. APP-CTF accumulates in TSPAN6-enriched microdomains, as demonstrated by sucrose gradient fractionation of CHAPSO-solubilized lysates from TSPAN6-overexpressing cells (Additional file 4: Figure S4a) and a proximity ligation assay of APP-CTF and myc-TSPAN6 (Additional file 4: Figure S4b). Interestingly, cells overexpressing TSPAN6 show larger ILVs (Fig. 4a-d), resembling those generated by the ESCRT-independent pathway [44]. We therefore compared the number of exosomes secreted from TSPAN6-overexpressing HEK293 cells and from control cells (Fig. 5d-g). We used the particle tracking system Nanosight to count and to determine the size of the vesicles present in the conditioned medium. TSPAN6 overexpression increased the average (μ) number of exosomes (μ_vector_ = 1280 × 10^6^; μ_Tspan6_ = 2139 × 10^6^; Fig. 5, f,g), which shows an average size of about 120 nm (Fig. 5 g). Both TSPAN6 and APP-CTF were present in exosomes isolated from HEK293 cells (Fig. 5e). Thus, TSPAN6 significantly affects the generation of exosomes, and although the amount of APP-CTF per exosome remains equal (Fig. 5e), the total pool of APP-CTF in exosomes is thus increased by 60%, partially explaining the APP-CTF accumulation in TSPAN6 overexpressing cells.

### The cytosolic adapter syntenin interacts with TSPAN6 and mediates the increased formation of ILVs fated for exosome secretion

The syndecan-syntenin-alix pathway triggers the biogenesis of ILVs destined to be secreted as exosomes, by-passing the degradative pathway [45]. These effects resemble those observed here in TSPAN6-overexpressing cells. In addition, syntenin interacts with the carboxy-terminal end of tetraspanin CD63 [39]. Thus we investigated if syntenin mediates the TSPAN6-induced generation of ILVs destined to exosomes and whether it might be involved in the decreased APP-CTF degradation.

**Fig. 6:**
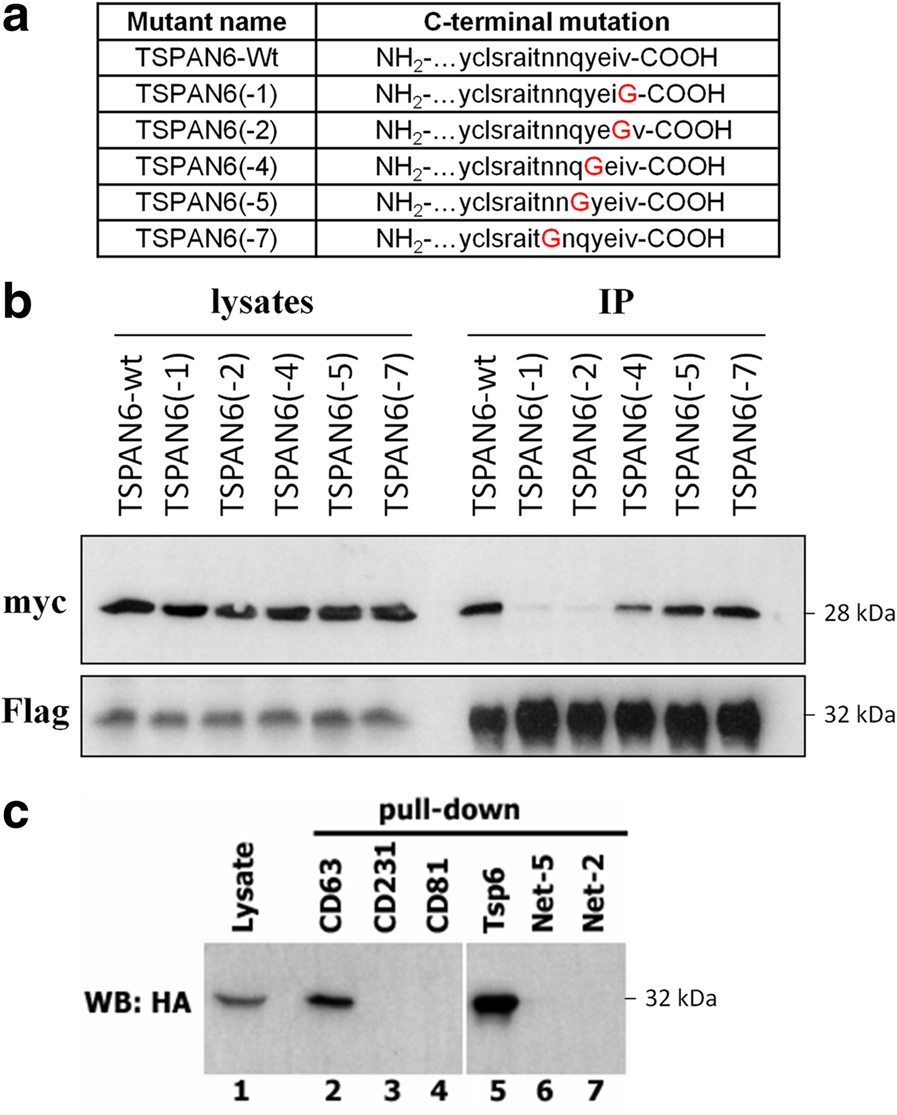
The C-terminal cytoplasmic region mediates the interaction of TSPAN6 with syntenin-1. **a** Table illustrating the location of the point mutations on the myc-tagged TSPAN6 mutants used in the co-immunoprecipitation experiment shown in **b. b** Co-expression of Flag-tagged syntenin in HEK293 cells together with myc-tagged TSPAN6-wt or lacking one of the last c-terminal amino acids (TSPAN6(-1) to TSPAN6(-7)). After immunoprecipitation of Flag-tagged syntenin, the interaction with the different TSPAN6 mutants was evaluated by western blot with an anti-myc antibody. The last 2 amino acids of TSPAN6 are essential for the interaction with syntenin. **c** N-terminally biotinylated peptides corresponding to the indicated C-termini of tetraspanins were synthesized and conjugated to avidin-agarose beads. HEK293 cells ectopically expressing HA-tagged syntenin were lysed in immunoprecipitation buffer (0.5% Brij 98-0.5% Triton X-100-PBS), incubated with the avidin-immobilized peptides and the interaction to syntenin was analyzed by western blot. TSPAN6 (Tsp6) and CD63 (positive control) C-terminal peptides also interacted with syntenin. Net-5, Net 2 (negative controls reported not to interact to syntenin), CD231 or CD81 did not interact to syntenin

Syntenin is an adapter protein that consists of an N-terminal domain, followed by a tandem PDZ module and a C-terminal domain. The PDZ module recognizes short sequences at the C-terminus of transmembrane proteins [46]. We investigated if the C-terminus of TSPAN6 contains a canonical PDZ-binding motif that can interact to syntenin. We therefore generated a series of mutants at the C-terminus of myc-tagged TSPAN6 (Fig. 6a) substituting each time a single amino acid residue for glycine. While Flag-tagged syntenin co-immunoprecipitates myc-TSPAN6, the substitution of one of the last two C-terminal amino acids by glycine disrupted this interaction (Fig. 6a,b). In addition we found that the immobilized C-terminal peptide of TSPAN6 and CD63 (positive control) but not the corresponding regions of some other tetraspanins (i.e., CD81, Net-5, Net-2 and CD231) can retain syntenin from cell lysates (Fig. 6c), confirming the specificity of this interaction.

**Fig. 7:**
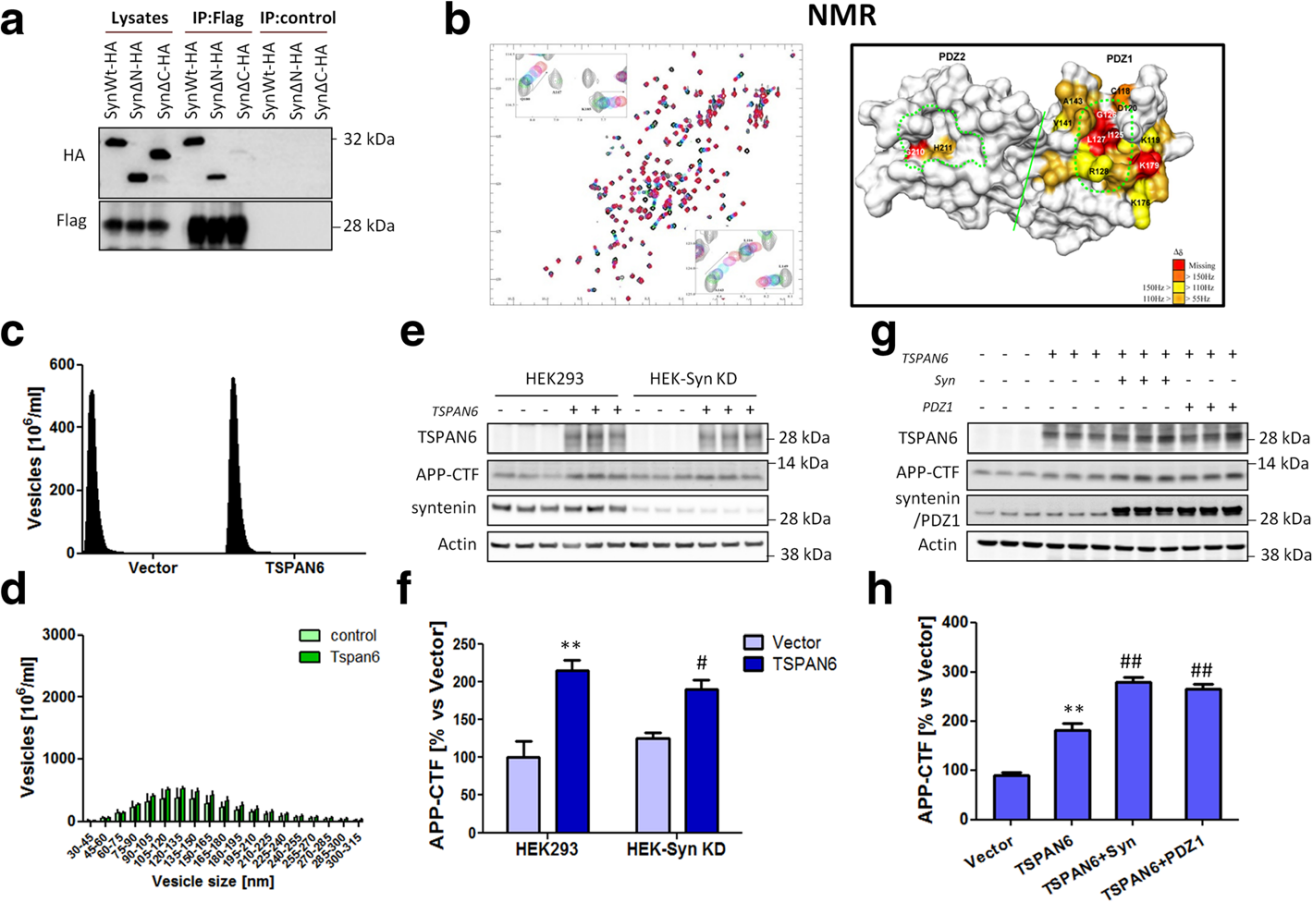
The interaction of syntenin with TSPAN6 mediates the enhanced formation of exosomes. **a** Flag-tagged TSPAN6 (Flag-TSPAN6) and HA-tagged syntenin (SynWt-HA) or syntenin lacking either the N-terminal fragment (SynΔN-HA) or the C-terminal fragment (SynΔC-HA) were co-transfected in HEK293 cells. Flag-TSPAN6 was immunoprecipitated with an anti-Flag antibody from cell lysates and the interaction was evaluated by western blot using an anti-HA antibody. Total lysates (first 3 lanes) show that the 3 syntenin constructs were expressed. However only SynWt-HA and SynΔN-HA but not SynΔC-HA were immunoprecipitated with Flag-TSPAN6. The absence of anti-Flag antibody gives no bands (negative control, last 3 lanes). **b** Nuclear magnetic resonance spectroscopy (NMR) analysis of syntenin-1 and TSPAN6 interaction. The left panel shows the superposition of six two-dimensional 1H-15 N-HSQC spectra of uniformly 15 N-labeled syntenin-1 PDZ domain (200 μM) titrated with increasing concentrations of C-terminal peptide of TSPAN6. The right panel shows the locations of syntenin PDZ residues involved in binding. **c** Exosomes isolated from the conditioned medium of HEK293 cells stably knocked down for syntenin (HEK-Syn KD) and overexpressing an empty vector (control) or TSPAN6 were analyzed by Nanosight. Overall, the number of exosomes is lower in HEK-Syn KD cells compared to HEK293 wt cells (Fig. 5**f**). Overexpression of TSPAN6 in HEK-Syn KD did not alter the number of exosomes secreted. **d** Same plot as in Fig. 5 **g** but for HEK293 cells stably knocked down for syntenin (HEK293-KD Syntenin). Neither number of exosomes nor size distribution change between TSPAN6-overexpressing and control cells (data from panel **c**). **e** HEK293 or HEK-Syn KD were co-transfected with an empty vector (control) or with TSPAN6 (*n* = 3 technical replicates). TSPAN6 overexpression increases the APP-CTF levels in both HEK293 and HEK-Syn KD cells. **f** Quantification of the western blots of panel **e. g** HEK293 cells were transfected with an empty vector (control), TSPAN6 alone or with either syntenin (Syn) or syntenin with a point mutation in PDZ1 domain (PDZ1) that prevents the interaction to TSPAN6. Lysates were analyzed by western blot (*n* = 3 technical replicates). Overexpression of TSPAN6 increases the APP-CTF levels. This effect is potentiated by co-expression of Syn or PDZ1 mutant. **h** Quantification of APP-CTF levels from the western blot shown in panel **g**. Statistical analysis was carried out with *t*-test (** *p* <0.01 vs vector; ## p0.01;vs TSPAN6 alone)

To further demonstrate the interaction between TSPAN6 and syntenin in vitro, we co-transfected HEK293 cells with Flag-TSPAN6 and HA-tagged wt syntenin (SYNwt-HA) or HA-tagged syntenin lacking the first 101 N-terminal (SYNΔN-HA) or the last 17 C-terminal amino acids (SYNΔC-HA). Flag-TSPAN6 immunoprecipitated SYNwt-HA and SYNΔN-HA but not SYNΔC-HA, suggesting that, like CD63, the C-terminus of syntenin is necessary for the stabilization of the interaction with TSPAN6 (Fig. 7a). In order to investigate the role played by the PDZ1 and PDZ2 domains of syntenin in the interaction, we performed NMR titration analysis of ^15^N-labeled syntenin PDZ1&2 with unlabeled C-terminal peptide of TSPAN6 consisting of the last 10 amino acids of the human protein (TSPAN6-C). The chemical shift perturbations were used to map the binding sites, revealing a contact surface for TSPAN6-C predominantly in the PDZ1 domain binding pocket (Fig. 7b).

In order to evaluate the relevance of the TSPAN6-syntenin interaction for exosome production, we compared the number of these vesicles secreted by HEK293 cells after stable knock down of syntenin (HEK-Syn KD) and transfected with an empty vector or with a vector expressing TSPAN6 (Fig. 7c). The total number of exosomes generated by HEK-Syn KD is dramatically lower compared to the wild type HEK293 cells, in agreement with the role that syntenin, together with syndecan and alix, plays in the biogenesis of exosomes [45]. Interestingly, in HEK-Syn KD cells, and as opposed to HEK293 wt cells (Fig. 5f,g), overexpression of TSPAN6 did not increase the total number of exosomes (compare Fig. 5f,g with Fig. 7c), nor the size distribution (Fig. 7d). This clearly suggests that the TSPAN6-syntenin interaction is necessary in the formation of ILVs destined to exosomes. We next investigated whether TSPAN6-triggered accumulation of APP-CTF occurs in HEK-Syn KD cells. The western blot analysis shows that TSPAN6 can induce the same APP-CTF accumulation in both wt and KD cells (Fig. 7e,f), suggesting that syntenin is largely dispensable for the effect. This conclusion was supported by the observation that the same amount of APP-CTF accumulated in HEK293 after co-expressing TSPAN6 together with syntenin wt (Syn-wt) or a syntenin mutant which is unable to interact with TSPAN6 (Syn-PDZ1*) (Fig. 7 g,h). The results thus suggest that TSPAN6 stabilizes APP-CTF by an additional mechanism upstream of syntenin-induced exosome formation.

### TSPAN6 overexpression impairs the fusion of autophagosomes to lysosomes

**Fig. 8:**
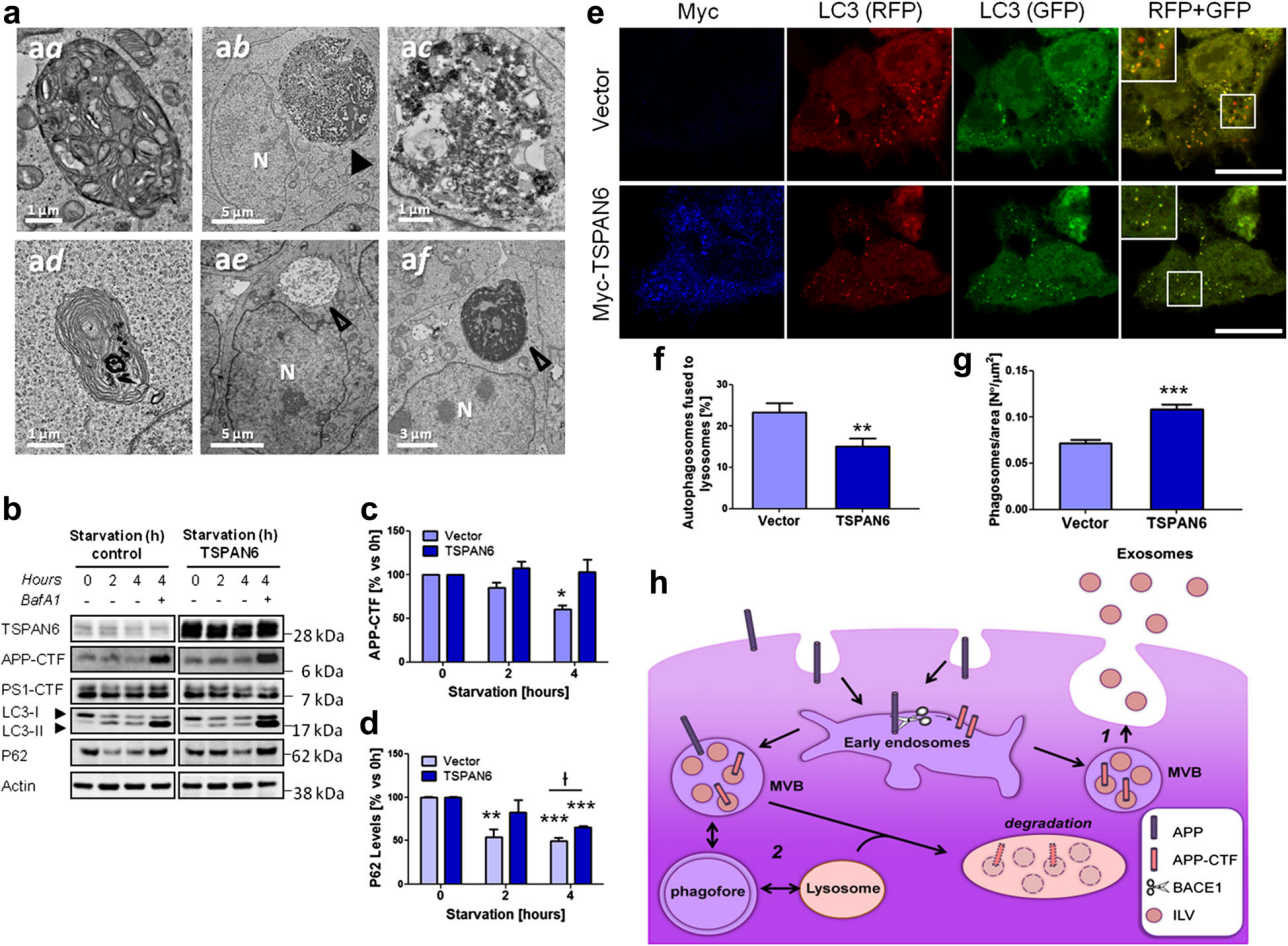
Overexpression of TSPAN6 compromises autophagy. **a** HEK293 cells overexpressing TSPAN6 analyzed by TEM show phagosomes (a*a*) multilamellar bodies (a*d*) and large vacuoles containing undegraded heterogeneous material in the lumen (a*b*-a*c,* arrowheads). In comparison, HEK293 cells expressing an empty vector present a reduced number of enlarged vesicles with homogeneous luminal material (a*e*-a*f*, empty arrowheads). Nucleus is indicated with an N. **b** Representative experiment showing autophagy induction in control (empty vector) or TSPAN6-overexpressing HEK293 cells by 2 h and 4 h starvation. Lysates were analyzed by western blot with antibodies against TSPAN6, APP, P62, LC3β, PS1-CTF and actin. TSPAN6-overexpressing cells show no degradation of APP-CTF after 4 h starvation compared to control cells. Activation of autophagy was assessed by conversion of LC3-I into LC3-II and the degradation of P62. The levels of PS1-CTF and actin were used as negative controls. **c** Quantification of APP-CTF levels from western blots like in panel **b** (*n* = 3 experiments). **d** Quantification of P62 levels from western blots like in panel **b** (*n* = 3 experiments). **e** HEK293 cells co-transfected with LC3-GFP-RFP and an empty vector or myc-TSPAN6 were starved for 3 h to induce autophagy. The fusion of autophagosomes (red and green) with lysosomes (red) is reflected in the loss of the fluorescence of GFP protein due to the low pH found in lysosomes. TSPAN6 overexpression is detected with an anti-myc antibody (blue) (scale bar = 10 μm). **f** Ratio between the number of autophagosomes fused to lysosomes versus the total number of autophagosomes (2 independent experiment; *n* = 43 autophagosomes for control; *n* = 43 autophagosomes for TSPAN6). **g** The number of phagosomes not fused to lysosomes (red and green) per cell is increased in cells overexpressing TSPAN6 (2 independent experiments, *n* = 59 cells for control; *n* = 80 cells for TSPAN6). **h** Scheme showing the cellular pathways affected by TSPAN6. 1-Increased levels of TSPAN6 increment the secretion of exosomes by a syntenin-dependent mechanism. 2-TSPAN6 impairs the fusion between autophagosomes and lysosomes affecting the cellular proteostasis. Statistical significance was determined by t- test (*p <0.05, **p <0.01, ***p <0.001), Ɨ *p* <0.05)

TSPAN6 induces the accumulation of enlarged endosomal compartments (Fig. 4a-c) suggesting an impairment of the downstream degradative pathways. In addition we found that autophagosomal and multilamellar compartments, (Fig. 8aa-ad) together with enlarged endosomes filled with heterogeneous material (Fig. 8ab-ac) are more commonly found in HEK293 cells overexpressing TSPAN6.

Hence we investigated whether TSPAN6 overexpression affects the degradation of APP-CTF by impairing the autolysosomal pathway. Firstly, we determined how the autophagic flux affects APP-CTF degradation in non-transfected (control) or TSPAN6-transfected HEK293 cells (Fig. 8b-d). Autophagy was induced by starvation of cells cultured in serum free Earle's Balanced Salt Solution (EBSS) medium for 2 h and 4 h, as previously reported [47–49]. The lysosome inhibitor bafilomycin A1 was added to the medium of 4 h starved cells as a positive control to estimate maximal APP-CTF accumulation. In control cells, APP-CTF levels decreased by about 40% after 4 h of starvation while the levels remained unchanged in TSPAN6-overexpressing cells (Fig. 8b,c). The levels of p62, a protein involved in the recognition of ubiquitinated proteins by the autophagosome, were also less decreased during autophagy induction in TSPAN6-overexpressing cells (Fig. 8b-d), though the impairment on p62 degradation was not so pronounced as for APP-CTF.

In order to test the hypothesis that TSPAN6 impairs the fusion between autophagosomes and lysosomes, HEK293 cells were co-transfected with the autophagosome-associated protein LC3 double-tagged with GFP and RFP (LC3-GFP-RFP) and an empty vector or myc-TSPAN6. Cells were put into starvation with EBBS medium for 3 h to induce the formation of autophagosomes (Fig. 8e). The fusion of newly formed autophagosomes with lysosomes is reflected in loss of green fluorescence of the GFP protein due to the low pH of lysosomes. Cells overexpressing myc-TSPAN6 (blue) showed a decreased ratio of the number of red vesicles versus the total number of vesicles (red and green) per cell (Fig. 8f). This, together with the parallel increased number of autophagosomes in cells overexpressing myc-TSPAN6 (Fig. 8 g), suggests a decreased fusion of autophagosomes to lysosomes. Cells kept in normal growth medium in similar circumstances did not display autophagosomes (Additional file 5: Figure S5).

### APP processing is altered in neurons of *Tspan6* KO mice

**Fig. 9:**
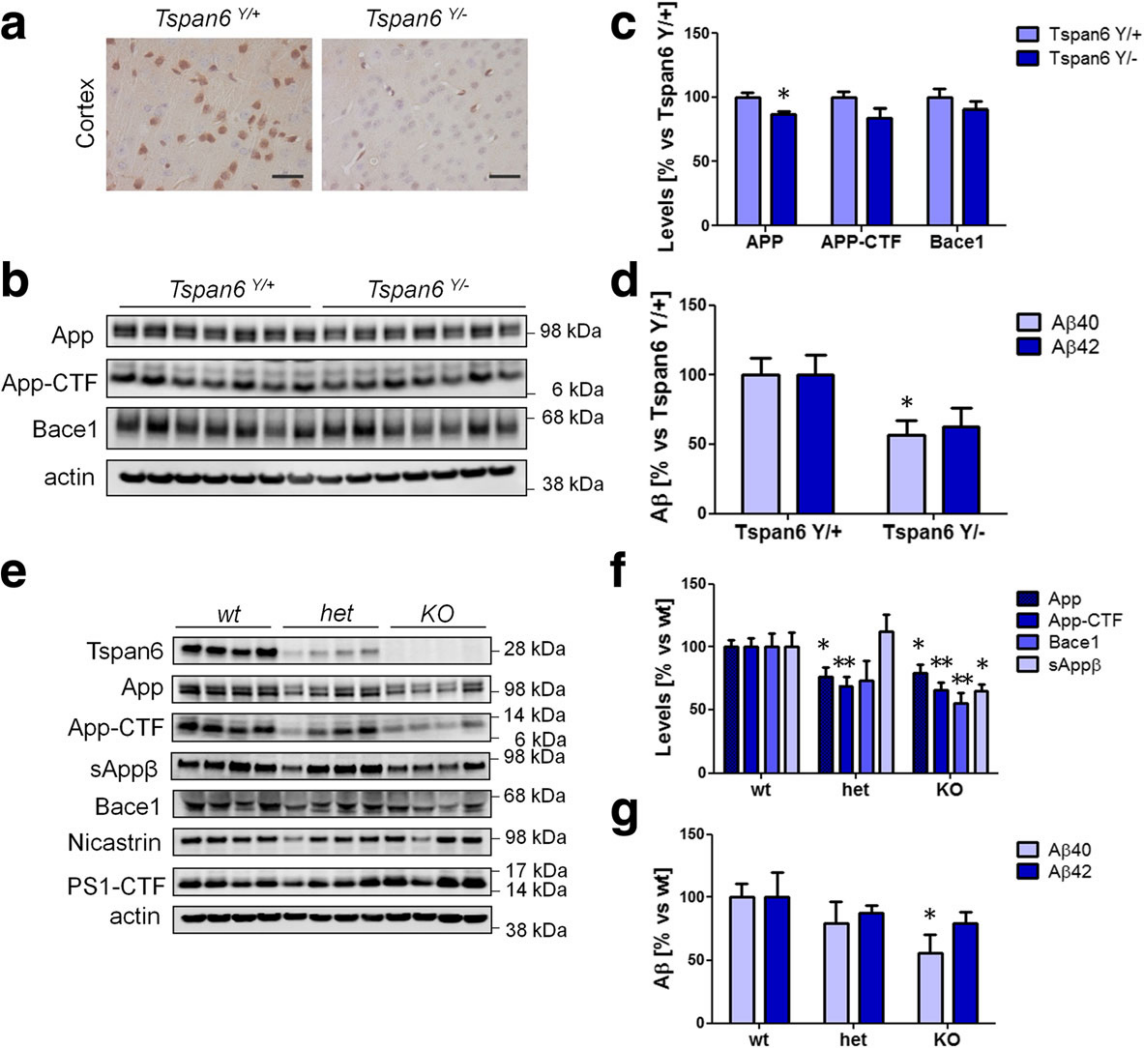
TSPAN6 controls neural protein homeostasis in vivo. **a** Immunohistochemistry on cerebral sections of the cortex (layer 4) of *Tspan6* ^*Y/+*^ and *Tspan6* ^*Y/-*^ adult mice (1 year old, scale bar = 40 μm). Sections were probed with a polyclonal anti-Tspan6 antibody. Only neuronal bodies of Tspan6 ^*Y/+*^ were positive. **b** Western blot analysis of the cerebral cortex of *Tspan6* ^*Y/+*^ and *Tspan6* ^*Y/-*^ adult mice (7 x *Tspan6* ^*Y/+*^ and 7 x *Tspan6* ^*Y/-*^ 1 year old mice). **c** Quantification of bands shown in panel **b** shows a statistically significant decrease of the App levels in *Tspan6* ^*Y/-*^ mice and slightly decreased levels of App-CTF and Bace1 that do not reach statistical significance. **d** ELISA determinations of the Aβ content in the cerebral cortex of the mice in panel **b**. Aβ_40_ levels were significantly decreased in *Tspan6* ^*Y/-*^ mice. **e** Western blot analysis of lysates of *Tspan6 wt (Tspan6* ^*+/+*^ *or Tspan6* ^*Y/+*^ *)* ^,^ *Tspan6 het* (*Tspan6* ^*+/-*^) and *Tspan6 KO* (*Tspan6* ^*-/-*^ or *Tspan6* ^*Y/-*^) E15 primary neuronal cultures showing a reduction in the protein levels of App, App-CTF, sAppβ and Bace1 in *Tspan6 het* and *KO* cultures. **f** Quantification of the intensity of the bands of western blot experiments as in panel **e** show a statistically significant reduction in the protein levels of App, App-CTF, sAppβ and Bace1 in *Tspan6 KO* primary neuronal cultures (*n* = 8 for *wt*, *n* = 8 for *het*, *n* = 8 for *KO*). **g** Aβ levels measured by ELISA in 24 h conditioned medium show a statistical significant reduction of Aβ_40_ levels in *Tspan 6 KO* neurons (*n* = 8 for *wt*, n = 3 for *het*, and *n* = 4 for *KO*). Statistical significance was determined by *t*-test (*p <0.05, **p <0.01)

**Fig. 10:**
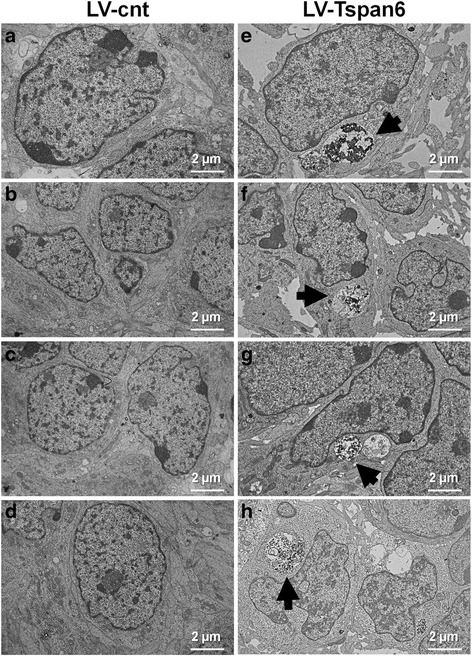
6 DIV primary cortical neurons derived from *Tspan6 wt* (*Tspan6* ^*+/Y*^) E14.5 embryos were infected with an empty lentiviral vector (LV-cnt) or a lentiviral vector overexpressing Tspan6 (LV-Tspan6). At 9 DIV neurons were washed and processed for EM analysis. *Tspan6 wt* neurons that overexpressed Tspan6 (**e-h**) show alterations by EM consisting of accumulation of electron dense material inside large vesicles (arrow) in the soma compared to control neurons (**a-d**)

In order to evaluate whether TSPAN6 exerts similar effects on the amyloidogenic pathway in a physiologically relevant context we investigated the effect of a loss of function mutation on APP processing in the cerebral cortex of adult *Tspan6 wt* or *Tspan6 KO* mice. As shown in Fig. 9a, Tspan6 is expressed in neuronal bodies of the cerebral cortex of *Tspan6 wt* mice but not in *Tspan6 KO* mice. We observed a moderate to intense Tspan6 reactivity in brain regions with neurons expressing glutamate decarboxylase (GAD)-67, an enzyme involved in GABA synthesis, and in neurons expressing the vesicular glutamate transporter 1 (vGLUT1), a glutamate transporter that transports cytoplasmic glutamate into vesicles (Additional file 6: Figure S6). We then compared the expression levels of App and Bace1 in the cerebral cortex of 1 year old *Tspan6 wt* and *Tspan6 KO* mice by western blot (Fig. 9b). App expression levels were significantly decreased in *Tspan6 KO* mice, while App-CTF and Bace1 showed minimal effects (Fig. 9b,c). *Tspan6 KO* mice show also lower levels of Aβ_40_ and Aβ_42_ peptides in the cerebral cortex (Fig. 9d). We also cultured primary cortical neurons from *Tspan6 wt*, *het,* or *KO* E14.5 embryos. The expression levels of App-CTFs were decreased in *Tspan6 het* and *Tspan6 KO neurons* compared to *Tspan6 wt* neurons (Fig. 9e,f). The protein levels of App, App-CTFs, Bace1, and the secretion of sAppβ were decreased in *Tspan6 KO* neurons (Fig. 9e, f). *Tspan6 KO* neurons produced less Aβ_40_ compared to *Tspan6 wt* neurons (Fig. 9 g), while Aβ_42_ was only minimally affected. Finally overexpression of Tspan6 in *Tspan6 wt* and *KO neurons* increased the protein levels of App and p62, indicating a deficit in autophagy, and the secretion of Aβ_40_ and Aβ_42_ to the media (Additional file 7: Figure S7a,b). However no effects on App-CTF and Bace1 levels were observed. The impairment of autophagy was confirmed by EM analysis of *Tspan6 wt* neurons overexpressing Tspan6, which show enlarged vesicles with undegraded material in the lumen (Fig. 10), resembling those found in TSPAN6-overexpressing HEK cells. These data demonstrate that Tspan6 is physiologically involved in neural APP metabolism.

Finally, we investigated the consequences of Tspan6 loss in an AD mouse model by crossing *Tspan6 KO* mice with mice expressing endogenous levels of the human APP gene with the Swedish and Iberian mutations (App^NL/F^) that trigger the formation of amyloid plaques [41]. When we examined by western blot total lysates of the cerebral cortex of 1 year old App^NL/F^ x *Tspan6 KO*, we observe a decrease in the levels of App-CTF, Bace1, Aβ and p62 in comparison to App^NL/F^ x *Tspan6 wt* mice. However we did not find differences in terms of amyloid plaque content by using a combination of a brain clearing methodology with Thioflavin-S staining (Additional file 8: Figure S8c).

## Discussion

We demonstrate here that TSPAN6 is localized in endosomal and lysosomal structures and plays a critical role in the complex housekeeping of MVBs in the cell. Overexpression of TSPAN6 dramatically altered the morphology of these structures, causing alterations in the turnover of several proteins including APP-CTF, BACE1 and Aβ involved in AD. The increased secretion of Aβ peptide seems to be a direct consequence of the increment in the levels of APP-CTF due to TSPAN6 overexpression, as supported by the parallel increase in the levels of secreted Aβ and transfected FlagAPP-C99 in cells overexpressing TSPAN6. This was corroborated in TSPAN6-overexpressing primary neurons, were an accumulation of transduced FlagAPP-C99 is observed (Additional file 9: Figure S9). On the other hand we could not demonstrate APP-CTF accumulation at endogeneous levels of APP expression in neurons, in contrast to HEK cells. Apparently, as we see still increased Aβ secretion in TSPAN6 overexpressing neurons, the neurons have additional capacity to turn-over APP-CTF. (Additional file 7: Figure S7b).

It is clear that the effects of TSPAN6 on APP-CTF and Aβ generation are complex. Apart from a decreased turn-over (which makes more APP-CTF available for γ-secretase cleavage), and an increased secretion of APP-CTF containing exosomes, there is also an increase of BACE1 levels by TSPAN6 in HEK 293 cells. While this also might contribute to the increased generation of secreted Aβ, the fact that APP-CTFs are stabilized when overexpressed, indicates a BACE-1 independent mechanism.

The effect of TSPAN6 on APP-CTF, decreasing its degradation and increasing exosome release, suggests that TSPAN6 affects the lysosomal-MVB-exosome system at the level of ILVs destined either for degradation or biogenesis of exosomes. The sorting of proteins to ILVs is a crucial step in the proper degradation of proteins (including APP) as MVBs fuse to lysosomes but also determines the secretion of proteins via exosomes. At least two molecular signals have been involved in determining the type of ILVs generated: the ESCRT-dependent pathway drives the degradation of the cargo via lysosomes/autophagosomes while ESCRT-independent pathways, such as ceramide-rich membrane microdomains or tetraspanins, are thought to generate ILVs destined to exosomes. Our results show that overexpression of TSPAN6 in HEK293 cells induces enlarged endosomes with ILVs morphologically resembling those of ESCRT-depleted cells. In addition this is accompanied by an increased secretion of exosomes which depends on the specific interaction of TSPAN6 with syntenin. Syntenin allows syndecans and associated molecules to escape degradation by facilitating their translocation to the exosomal route [45]. Thus, our data suggest that TSPAN6 induces an ESCRT-independent formation of ILVs destined for the biogenesis of exosomes in disfavor of an ESCRT-dependent pathway destined to degradation, which explains the stabilization of APP-CTF. Although TSPAN6 interaction with syntenin is required for the increased formation of exosomes, we found that TSPAN6 overexpression increased APP-CTF levels even in the absence of syntenin. These results suggest that TSPAN6 exerts an additional proteostatic effect on APP-CTF. However, it is interesting to notice that in low syntenin conditions, TSPAN6-triggered APP-CTF accumulation is less pronounced, suggesting that syntenin is responsible for at least part of the effect observed. In turn, the PDZ mutant of syntenin can per se induce the accumulation of APP-CTF fragments, indicating the existence of a TSPAN6-independent role of syntenin in stabilizing APP-CTF.

The enhancement of ESCRT-independent mechanisms impairs autophagy by affecting the fusion between autophagosomes and lysosomes, as illustrated by the accumulation of autophagosomes in ESCRT-depleted cells. Interestingly, TSPAN6 appears in a recently published list of newly identified modulators of autophagy [28]. Here, we find decreased fusion between autophagosomes and lysosomes in cells overexpressing TSPAN6, accompanied by the accumulation of the autophagic marker p62 in HEK293 cells. The impairment of autophagy provides a mechanistic explanation for the accumulation of APP-CTF in cells overexpressing TSPAN6. In agreement with a pivotal role for TSPAN6 at this critical junction, a reduction in the levels of TSPAN6 triggers the opposite effect of overexpression: a decrease in the levels of APP-CTF in HEK cells and in neurons. However, further work is needed to support an impairment of the ESCRT-pathway by TSPAN6.

Interestingly, alterations in the endolysosomal–autophagic system are well-recognized early pathological features of AD, which is marked by prominent enlargement of endosomal compartments and progressive accumulation of autophagic vacuoles [50]. In addition, neuronal deficiency of *presenilin 1*, whose mutations trigger the dominant form of the disease [51], causes accumulation of autophagosomes and MVBs [52, 53], while conditional knockout mice for *presenilin 1* accumulate autophagosomes in the brain [54], indicating a link between abnormal processing of presenilin substrates and abnormalities in those pathways. By co-expressing the Rab5^Q79L^ mutant with TSPAN6 in HEK293 cells, we observe exacerbated enlarged Rab5-positive vesicles similar to those seen in neurons generated from induced pluripotent stem cells from AD patients and Down syndrome fibroblasts [55, 56]. Elevated levels of the APP-βCTF fragment generated by BACE1 are sufficient to trigger the accumulation of swollen endosomes by recruiting APPL1 [57]. Since BACE1 protein levels and the APP-βCTF fragment are increased in TSPAN6-overexpressing cells, it is plausible that the formation and accumulation of enlarged endosomes are the consequence of APP-βCTF accumulation. Further work should address to what extent the accumulating APP-CTF may contribute to some of these alterations. Further work is also needed to explain the effects of TSPAN6 on the processivity of γ-secretase processing reflected in increased Aβ_40._ As the effect is not observed in cell free γ-secretase assays, it is likely indirect and possibly caused by redistribution of APP-CTF processing over different subcellular compartments or even different γ-secretase complexes [58] after TSPAN6 overexpression, however, this needs further investigation. On the other hand, the fact that the decreased expression levels of soluble Aβ and proteins involved in the amyloidogenic pathway observed in App^NL/F^ x *Tspan6 KO* mice do not translate into a lower number of plaques in these animals, suggests that Tspan6 may be involved in other biological processes controlling Aβ accumulation in the brain such as glial-dependent Aβ uptake or clearance of the peptide from the brain through the blood brain barrier. Further studies should clarify these other potential roles.

## Conclusions

In conclusion, our study identifies TSPAN6, a moderately up-regulated gene in the prefrontal cortex of AD patients, as a new modulator of APP-CTF proteostasis preventing its degradation by the impairment of the autolysosomal pathway, leading to consequent enhanced Aβ production, and turning the balance towards the formation of ILVs destined to form exosomes (Fig. 8 h). Human neurodegenerative diseases including Alzheimer's (AD), Huntington's, and Parkinson's diseases are associated with aberrant protein aggregation [59, 60], and loss of proteostatic mechanisms are likely very central in these diseases. Altogether, our work describes here the biological role of TSPAN6 in the MVB pathway and reinforces the idea that altered proteostasis in neurons may contribute to sporadic AD.

## Additional files

Additional file 1: Figure S1. a TSPAN6 was transfected in HEK293 cells stably expressing human wtAPP (HEK-APPwt; 3 independent experiments per condition) and analyzed by western blot using antibodies against ADAM10, BACE1 and components of the γ-secretase complex. BACE1 levels were increased while γ-secretase components are not affected. A representative actin blot of the samples (run on a separate gel) is shown. b The activity of α- and β-secretase was determined by measuring the sAPPα and sAPPβ levels by western blot, respectively, in the 24 h-conditioned medium of the HEK293-APPwt cells from panel a. c The quantification of the intensity of the bands in panel a and b shows that BACE1 and sAPPβ levels are enhanced by TSPAN6 overexpression (*n* = 3 per condition) without any observable effect on sAPPα. d Same samples used in Fig. 1d were analyzed by western blot as in panel a. Briefly, HEK-APPwt cells were treated with a scrambled siRNA (*siRNA-cnt*) or a siRNA against *TSPAN6* (*siRNA*-*TSPAN6*). A polyclonal TSPAN6 antibody was used to corroborate the decrease of TSPAN6 in siRNA-*TSPAN6* treated cells. BACE1 levels were strongly reduced in *siRNA*-*TSPAN6*-treated cells. A representative actin blot of the samples (run on a separate gel) is shown. e sAPPα and sAPPβ levels present in the 24 h-conditioned medium of cells of panel d were determined by western blot. f Quantification of the intensity of the bands in panel d and e shows that BACE1 and sAPPβ levels are decreased by TSPAN6 downregulation without any observable effect on sAPPα. Statistical significance was determined by *t* test (**p* <0.05, ***p* <0.01, ****p* <0.001). (PDF 92 kb)
Additional file 2: Figure S2. a Microsomal fractions from HEK293 cells downregulating (*siRNA-TSPAN6*; *siRNA-cnt* used as control; *n* = 3 technical repeats per condition) or overexpressing TSPAN6 (TSPAN6; empty vector used as control; *n* = 3 technical repeats per condition) were prepared for in vitro γ-secretase assay. The γ-secretase inhibitor DAPT (1 μM) was added as a negative control of the reaction. Analysis by western blot of the in vitro assays shows no effects on the production of AICD by downregulating or overexpressing TSPAN6. b In vitro γ-secretase assays were carried out by adding exogenous substrate (3xFlagAPP-C99) to microsomal fractions obtained from HEK293 cells overexpressing TSPAN6 or an empty vector (control) and analyzed by western blot. The γ-secretase inhibitor DAPT (1 μM) was added as a negative control. AICD production (detected with a monoclonal anti-Flag antibody) was not changed by overexpression of TSPAN6. The generation of different Aβ species during the in vitro reactions was determined by ELISA. No statistical differences in the different Aβ species produced were observed. c HEK293 cells treated with *siRNA-TSPAN6* or *siRNA-cnt* were transfected with the γ-secretase substrate myc-NΔE and analyzed by western blot (3 independent experiments). The blot was probed with a monoclonal anti-myc antibody to detect myc-NΔE, a monoclonal antibody against cleaved Notch1 (Val1744), a polyclonal antibody against TSPAN6 and a monoclonal antibody against actin. No effects on the myc-NΔE levels were observed when TSPAN6 is downregulated. d Plot of the ratio calculated from the bands in panel **c** between the γ-secretase-dependent generation of NICD and its substrate myc-NΔE. Cells down regulating TSPAN6 do not change the production of NICD. e Determination of *APP* mRNA levels of HEK293 cells transfected with an empty vector (control) or TSPAN6 by qPCR. No significant differences in the *APP* mRNA levels were observed between control or TSPAN6-overexpressing cells (*n* = 3 technical repeats per condition). f Determination of *BACE1* mRNA levels of HEK293 cells transfected with an empty vector (control) or TSPAN6 by qPCR. No significant differences in the *BACE1* mRNA levels were observed between control or TSPAN6-overexpressing cells (*n* = 3 technical repeats per condition). (PDF 182 kb)
Additional file 3: Figure S3. HEK293 cells transfected with myc-tagged TSPAN6 (myc-TSPAN6) were fixed and studied by immunofluorescence. Myc-TSPAN6, APP-CTF and APP-NTF were detected with a rat monoclonal antibody against myc, a rabbit monoclonal antibody against APP-CTF and a mouse monoclonal antibody against APP-NTF, respectively. Only APP-CTF shows a strong colocalization with myc-TSPAN6. Scale bar = 10 μM. (PDF 235 kb)
Additional file 4: Figure S4. a HEK293 cells were solubilized in 1% CHAPSO and fractionated by continuous sucrose gradient centrifugation. Fractions were collected and analyzed by western blot and probed with antibodies against markers for detergent-resistant (caveolin) or detergent-solubilized (calnexin) membranes, as well as APP, BACE1, γ-secretase components and TSPAN6. In the transfected cells APP-CTF showed a strong shift to the TSPAN6 positive fraction. The profile of the percentage of the intensity of the bands per each fraction versus the total intensity of APP-CTF and caveolin was plotted for control or TSPAN6-overexpressing cells. b Representative experiment of a proximity ligation assay (PLA) between APP-CTF and TSPAN6 on HEK293 cells transfected with an empty vector (control) or myc-tagged TSPAN6. Rabbit monoclonal antibody against APP-CTF (y188) and rat monoclonal antibody against myc were used as primary antibodies. Positive red dots indicating interaction events between APP-CTF and TSPAN6 were visualized by confocal microscopy, while cells expressing an empty vector were negative. (PDF 247 kb)
Additional file 5: Figure S5. Negative control of autophagosomes formation. HEK293 cells co-transfected with LC3-GFP-RFP and myc-tagged TSPAN6 (myc-TSPAN6) were left with culture medium containing 10% FBS. LC3-GFP-RFP was distributed over the cytosol in contrast to cells submitted to starvation (see Fig. 8e). Scale bars = 10 μm. (PDF 91 kb)
Additional file 6: Figure S6. Tspan6 immunoreactivity was present both in regions of high glutamatergic neuronal content (upper panels: GAD67, dentate gyrus) and high GABAergic neuronal content (lower panel: vGlut1, thalamic reticular nucleus). Scale bars = 18 μm. (PDF 119 kb)
Additional file 7: Figure S7. a Western blots of lysates of *Tspan6 wt* (*Tspan6 +/+* or *Tspan6 Y/+*) and *Tspan6 KO* (*Tspan6 -/-* or *Tspan6 Y/-*) primary neuronal cultures from different E14.5 embryos transduced with an empty lentiviral vector (LV-cnt) or a lentiviral vector overexpressing Tspan6 (LV-Tspan6). Overexpression of Tspan6 increases the levels of App and the autophagosomal marker p62, indicating a deficit in the autophagic pathway, but not of App-CTF and Bace1. Ponceau staining shows similar loading per well. b Increased levels of Aβ_40_ (upper panel) and Aβ_42_ (lower panel) determined by ELISA in the 12 h conditioned medium of *Tspan6 wt* (*Tspan6 +/+* or *Tspan6 Y/+*) and *Tspan6 KO* (*Tspan6 -/-* or *Tspan6 Y/-*) primary cortical neurons transduced with LV-Tspan6 (*n* = 12 different E14.5 embryos for *wt*, *n* = 9 different E14.5 embryos for *KO*) compared to neurons transduced with LV-cnt (*n* = 13 different E14.5 embryos for *wt*, *n* = 10 different E14.5 embryos for *KO*). Statistical significance was determined by *t* test (**p* <0.05, ***p* <0.01 and ****p* <0.001 for comparison between LV-cnt and LV-Tspan6 conditions, *Ɨ*p* <0.05 for comparison between *Tspan6 wt* and *Tspan6 KO*). (PDF 140 kb)
Additional file 8: Figure S8. a Western blot analysis of lysates from the cerebral cortex of 12 months old App^NL/F^ x *Tspan6 wt* (*n* = 5) and App^NL/F^ x *Tspan6 KO* (*n* = 6) mice. The membranes were blotted with antibodies against sAppβ, App, Aβ peptide, Bace1, p62 and actin. b Plot showing the quantification of the bands on the western blot of panel a. The levels of App-CTF, Bace1, Aβ and p62 are decreased in App^NL/F^ x *Tspan6 KO* mice (*n* = 6) compared to App^NL/F^ x *Tspan6 wt* mice (*n* = 5). c Thioflavin staining of cleared brains was performed from 1 year old App^NL/F^ x *Tspan6 wt* (*n* = 5) and App^NL/F^ x *Tspan6 KO* mice (*n* = 5) mice to visualize and quantify the 3D content of amyloid plaques. No differences in the number or the volume occupied by amyloid plaques was detected in App^NL/F^ x *Tspan6 KO* mice versus App^NL/F^ x *Tspan6 wt* mice. Statistical significance was determined by *t*-test (**p* <0.05, ***p* <0.01). (PDF 168 kb)
Additional file 9: Figure S9. a 6 DIV *Tspan6 wt* (*Tspan6 +/+* or *Tspan6 Y/+*), *Tspan6 het* (*Tspan6 -/+*) and *Tspan6 KO* (*Tspan6 -/- or Tspan6 Y/-*) primary neuronal cultures were transduced with an adenoviral vector expressing the human APP-C99 triple tagged with a Flag peptide (AV-3xFlagAPP-C99) at a MOI of 100. After 48 h, 4 independent cultures of each condition were lysed and analyzed by western blot using a monoclonal antibody against Flag in order to detect the FlagAPP-C99 expression; Actin is used as a loading control. b Quantification of bands shown in panel a. *Tspan6 KO* neurons accumulate less FlagAPP-C99 after transduction in comparison to *Tspan6 wt* neurons. Statistical significance was determined by *t*-test (**p* <0.05, ****p* <0.001). (PDF 106 kb)

## Abbreviations

AD: Alzheimer’s disease
APP: Amyloid precursor protein
APP-CTF: APP-C-terminal fragments
EBSS: Earle’s balanced salt solution
EE: Early endosomes
HEK293: Human embryonic kidney
ILVs: Intraluminal vesicles
MVBs: Multivesicular bodies
NICD: Notch intracelular domain
TSPAN6: Tetraspanin-6
