# Supplementary figures and images for: Tetraspanin 6: a pivotal protein of the multiple vesicular body determining exosome release and lysosomal degradation of amyloid precursor protein fragments
Source: Mol Neurodegener. 2017 Mar 10;12:25. doi: 10.1186/s13024-017-0165-0 (PMC5345265; doi:10.1186/s13024-017-0165-0)

**a**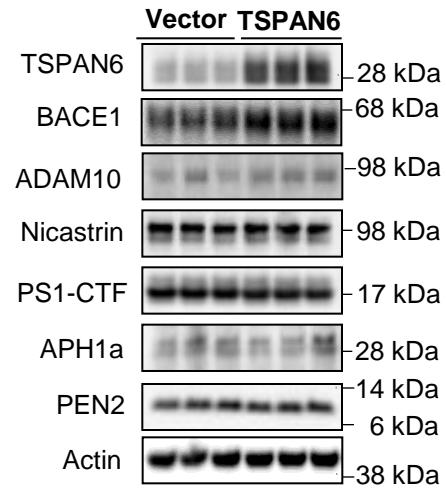**b**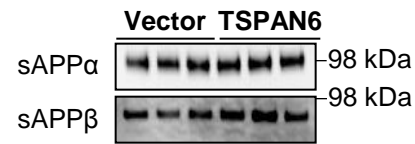**c**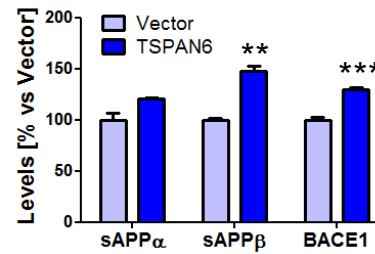**d**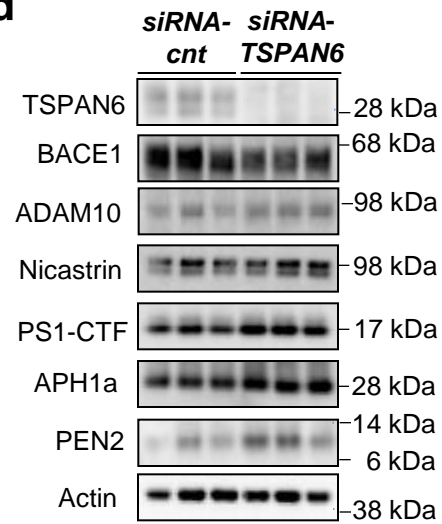**e**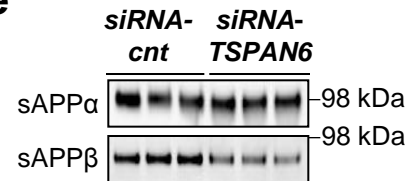**f**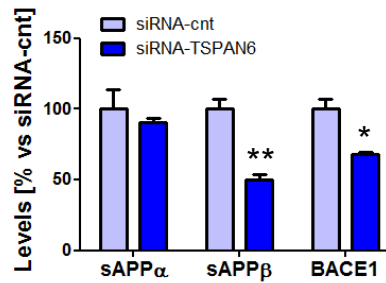

Supplement: Additional file 1: Figure S1. — a TSPAN6 was transfected in HEK293 cells stably expressing human wtAPP (HEK-APPwt; 3 independent experiments per condition) and analyzed by western blot using antibodies against ADAM10, BACE1 and components of the γ-secretase complex. BACE1 levels were increased while γ-secretase components are not affected. A representative actin blot of the samples (run on a separate gel) is shown. b The activity of α- and β-secretase was determined by measuring the sAPPα and sAPPβ levels by western blot, respectively, in the 24 h-conditioned medium of the HEK293-APPwt cells from panel a. c The quantification of the intensity of the bands in panel a and b shows that BACE1 and sAPPβ levels are enhanced by TSPAN6 overexpression (n = 3 per condition) without any observable effect on sAPPα. d Same samples used in Fig. 1d were analyzed by western blot as in panel a. Briefly, HEK-APPwt cells were treated with a scrambled siRNA (siRNA-cnt) or a siRNA against TSPAN6 (siRNA-TSPAN6). A polyclonal TSPAN6 antibody was used to corroborate the decrease of TSPAN6 in siRNA-TSPAN6 treated cells. BACE1 levels were strongly reduced in siRNA-TSPAN6-treated cells. A representative actin blot of the samples (run on a separate gel) is shown. e sAPPα and sAPPβ levels present in the 24 h-conditioned medium of cells of panel d were determined by western blot. f Quantification of the intensity of the bands in panel d and e shows that BACE1 and sAPPβ levels are decreased by TSPAN6 downregulation without any observable effect on sAPPα. Statistical significance was determined by t test (*p <0.05, **p <0.01, ***p <0.001). (PDF 92 kb) [file 13024_2017_165_MOESM1_ESM.pdf]

**a**

**Endogenous substrate**

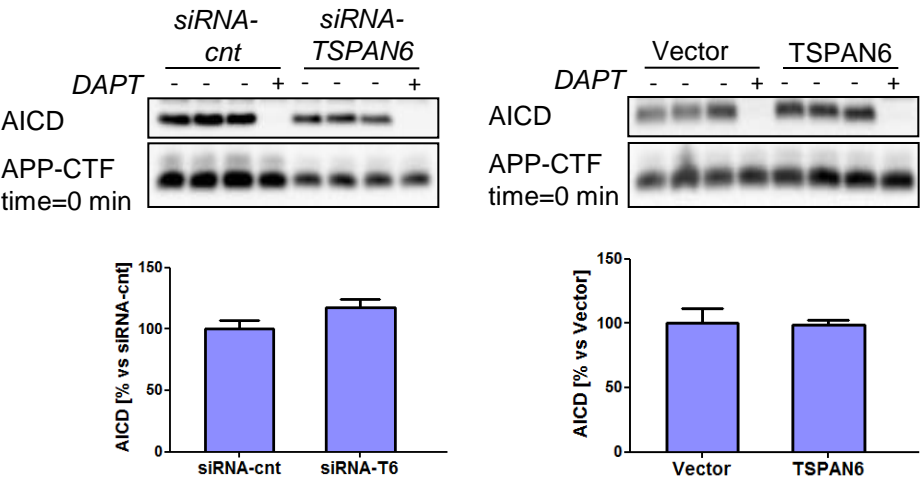

**b**

**Purified substrate**

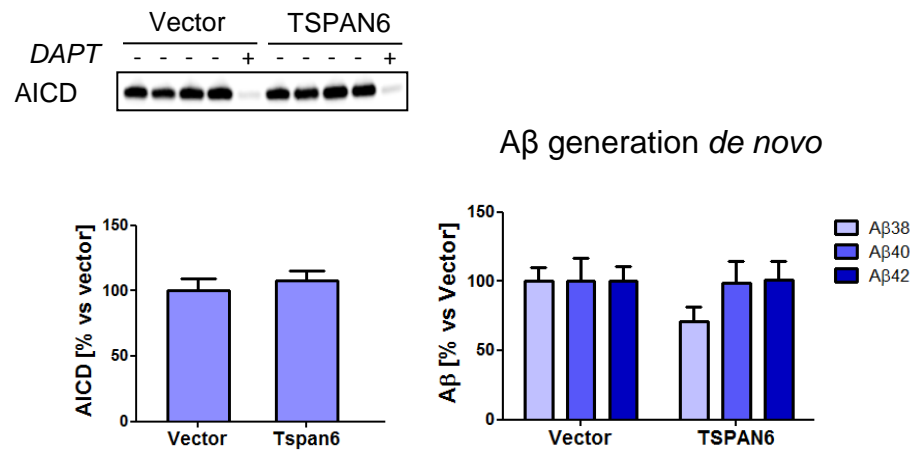

**c**

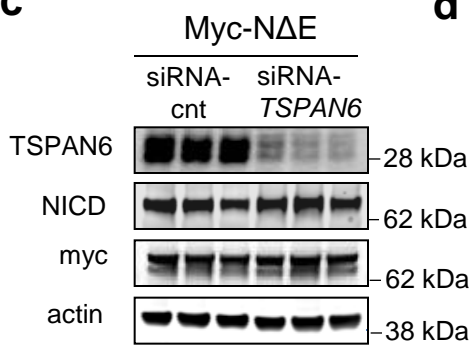

**d**

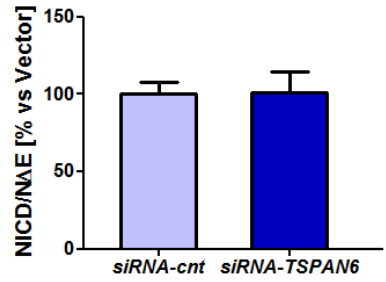

**e**

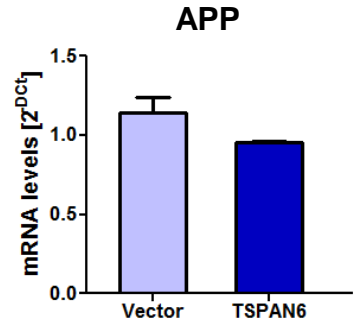

**f**

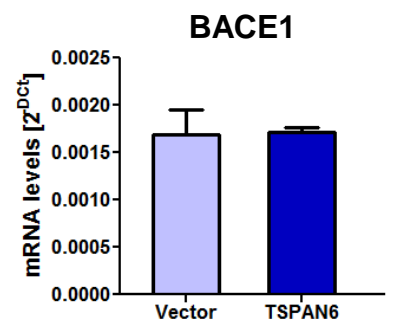

Supplement: Additional file 2: Figure S2. — a Microsomal fractions from HEK293 cells downregulating (siRNA-TSPAN6; siRNA-cnt used as control; n = 3 technical repeats per condition) or overexpressing TSPAN6 (TSPAN6; empty vector used as control; n = 3 technical repeats per condition) were prepared for in vitro γ-secretase assay. The γ-secretase inhibitor DAPT (1 μM) was added as a negative control of the reaction. Analysis by western blot of the in vitro assays shows no effects on the production of AICD by downregulating or overexpressing TSPAN6. b In vitro γ-secretase assays were carried out by adding exogenous substrate (3xFlagAPP-C99) to microsomal fractions obtained from HEK293 cells overexpressing TSPAN6 or an empty vector (control) and analyzed by western blot. The γ-secretase inhibitor DAPT (1 μM) was added as a negative control. AICD production (detected with a monoclonal anti-Flag antibody) was not changed by overexpression of TSPAN6. The generation of different Aβ species during the in vitro reactions was determined by ELISA. No statistical differences in the different Aβ species produced were observed. c HEK293 cells treated with siRNA-TSPAN6 or siRNA-cnt were transfected with the γ-secretase substrate myc-NΔE and analyzed by western blot (3 independent experiments). The blot was probed with a monoclonal anti-myc antibody to detect myc-NΔE, a monoclonal antibody against cleaved Notch1 (Val1744), a polyclonal antibody against TSPAN6 and a monoclonal antibody against actin. No effects on the myc-NΔE levels were observed when TSPAN6 is downregulated. d Plot of the ratio calculated from the bands in panel c between the γ-secretase-dependent generation of NICD and its substrate myc-NΔE. Cells down regulating TSPAN6 do not change the production of NICD. e Determination of APP mRNA levels of HEK293 cells transfected with an empty vector (control) or TSPAN6 by qPCR. No significant differences in the APP mRNA levels were observed between control or TSPAN6-overexpressing cells (n = 3 technical repeats per con [file 13024_2017_165_MOESM2_ESM.pdf]

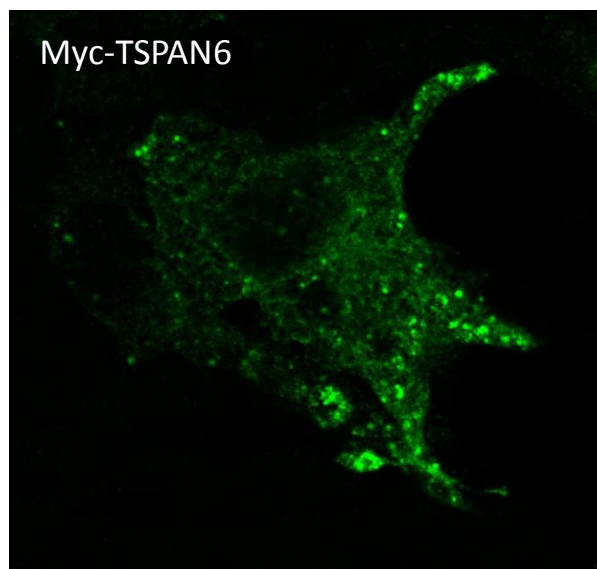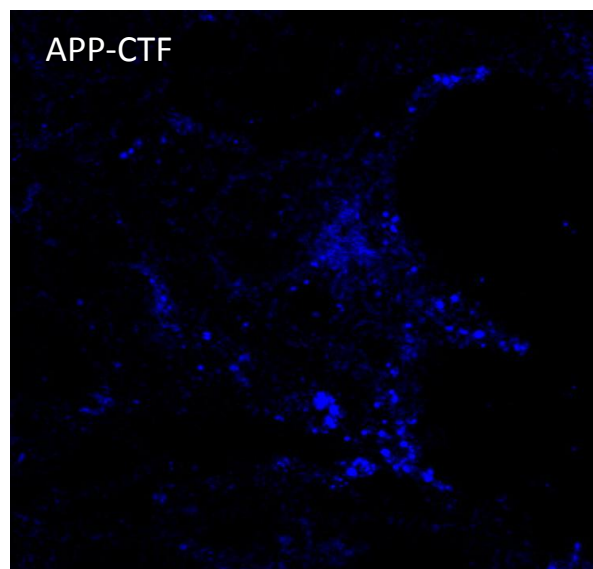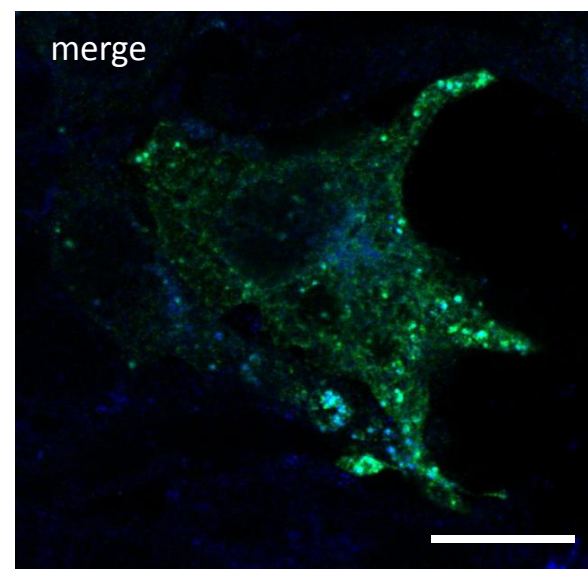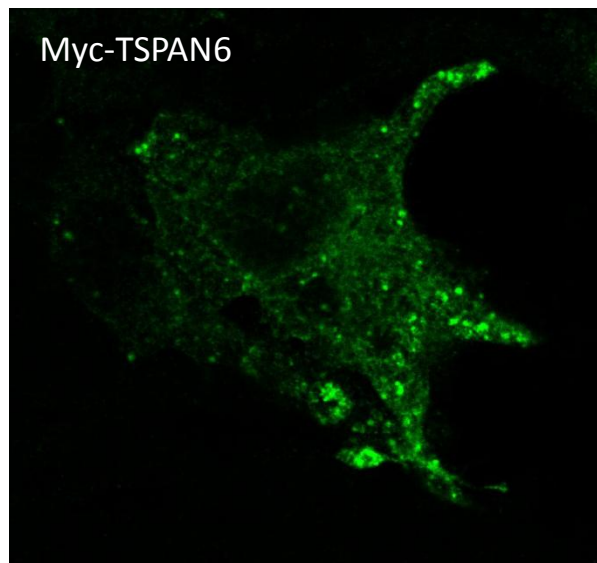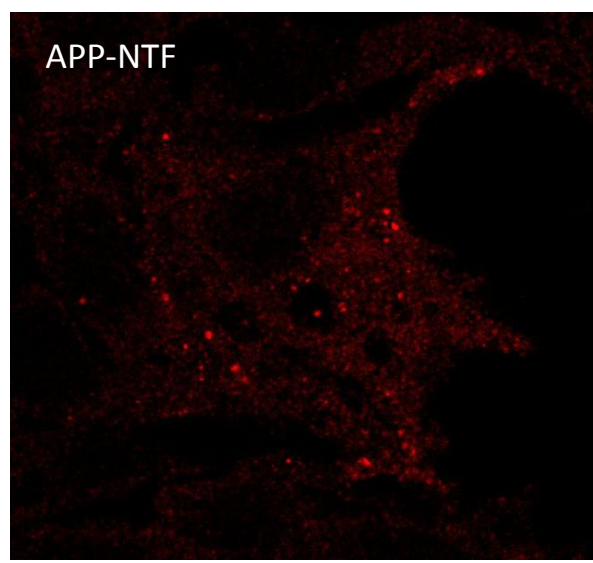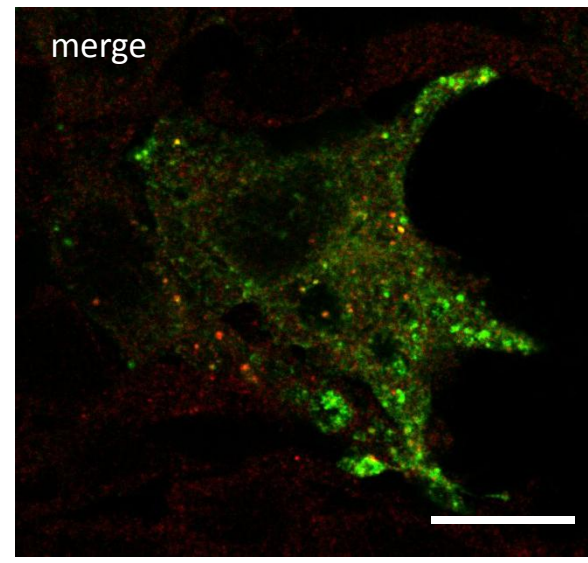

Supplement: Additional file 3: Figure S3. — HEK293 cells transfected with myc-tagged TSPAN6 (myc-TSPAN6) were fixed and studied by immunofluorescence. Myc-TSPAN6, APP-CTF and APP-NTF were detected with a rat monoclonal antibody against myc, a rabbit monoclonal antibody against APP-CTF and a mouse monoclonal antibody against APP-NTF, respectively. Only APP-CTF shows a strong colocalization with myc-TSPAN6. Scale bar = 10 μM. (PDF 235 kb) [file 13024_2017_165_MOESM3_ESM.pdf]

**a****Control****Myc-TSPAN6**

1 2 3 4 5 6 7 8 9 10 11 12

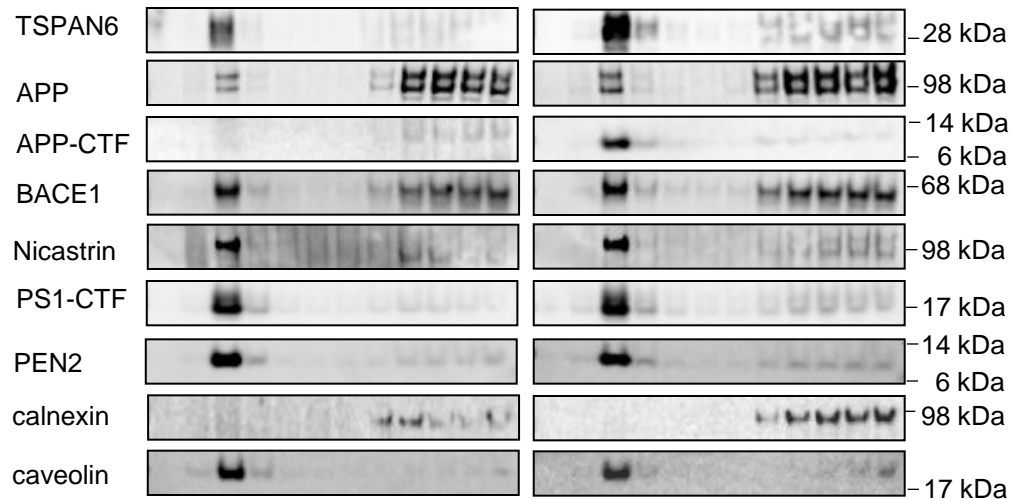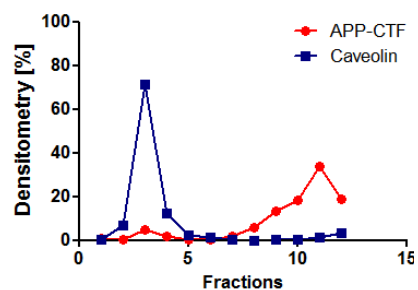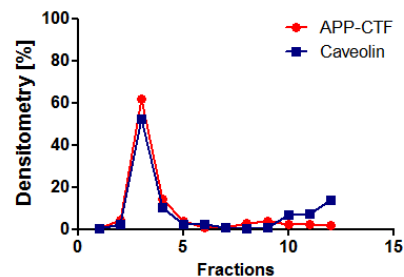**b****PLA assay**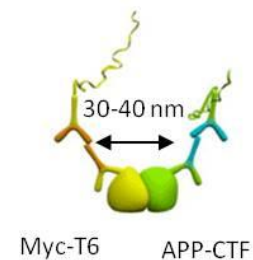**myc-TSPAN6****Vector**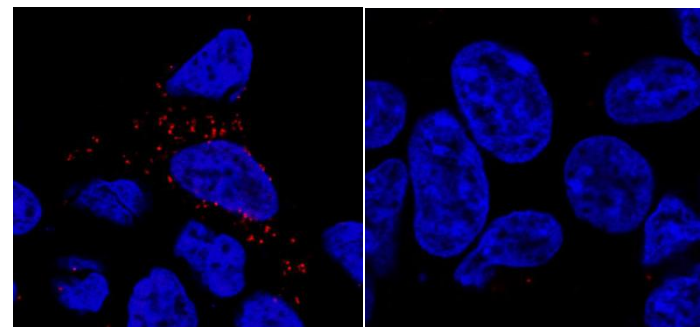

Supplement: Additional file 4: Figure S4. — a HEK293 cells were solubilized in 1% CHAPSO and fractionated by continuous sucrose gradient centrifugation. Fractions were collected and analyzed by western blot and probed with antibodies against markers for detergent-resistant (caveolin) or detergent-solubilized (calnexin) membranes, as well as APP, BACE1, γ-secretase components and TSPAN6. In the transfected cells APP-CTF showed a strong shift to the TSPAN6 positive fraction. The profile of the percentage of the intensity of the bands per each fraction versus the total intensity of APP-CTF and caveolin was plotted for control or TSPAN6-overexpressing cells. b Representative experiment of a proximity ligation assay (PLA) between APP-CTF and TSPAN6 on HEK293 cells transfected with an empty vector (control) or myc-tagged TSPAN6. Rabbit monoclonal antibody against APP-CTF (y188) and rat monoclonal antibody against myc were used as primary antibodies. Positive red dots indicating interaction events between APP-CTF and TSPAN6 were visualized by confocal microscopy, while cells expressing an empty vector were negative. (PDF 247 kb) [file 13024_2017_165_MOESM4_ESM.pdf]

## LC3-RFP-GFP

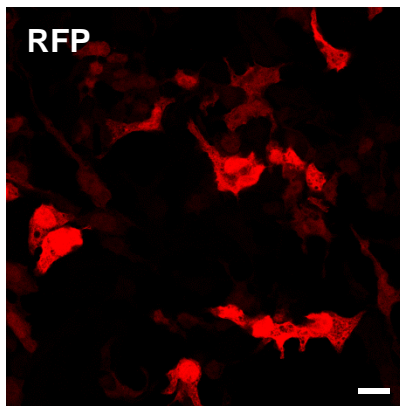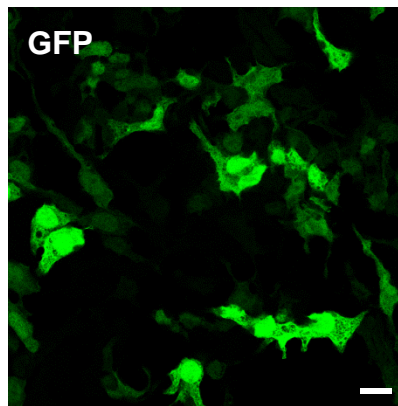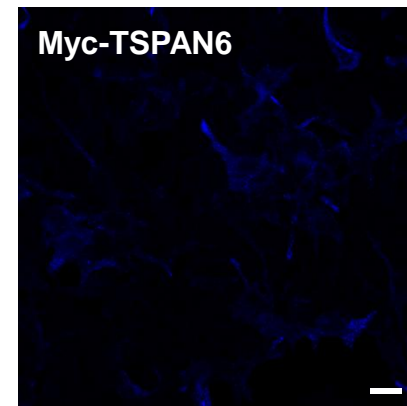

Supplement: Additional file 5: Figure S5. — Negative control of autophagosomes formation. HEK293 cells co-transfected with LC3-GFP-RFP and myc-tagged TSPAN6 (myc-TSPAN6) were left with culture medium containing 10% FBS. LC3-GFP-RFP was distributed over the cytosol in contrast to cells submitted to starvation (see Fig. 8e). Scale bars = 10 μm. (PDF 91 kb) [file 13024_2017_165_MOESM5_ESM.pdf]

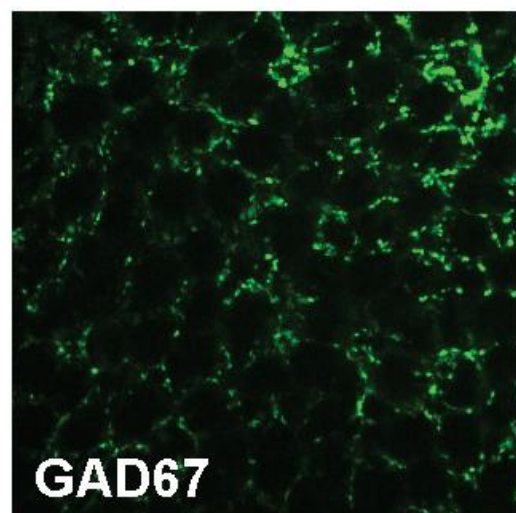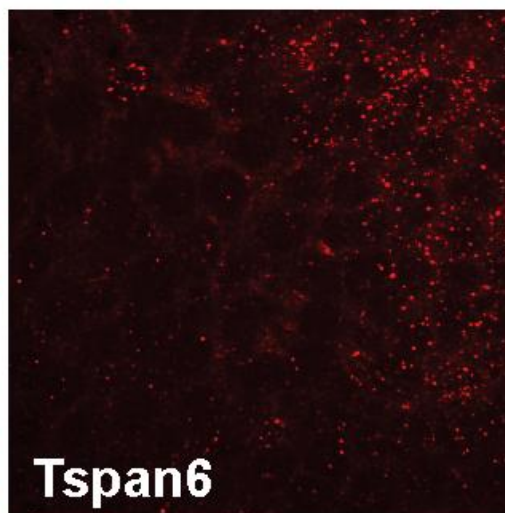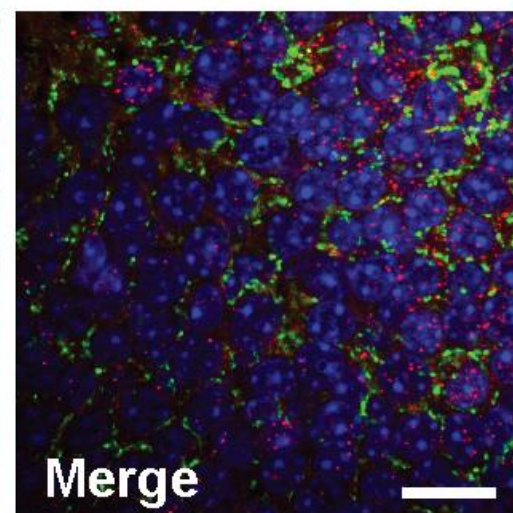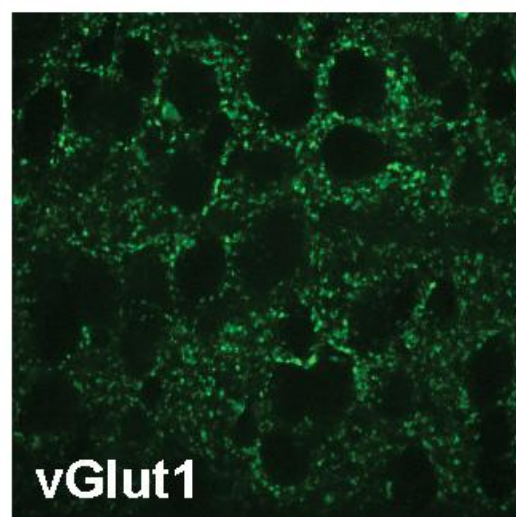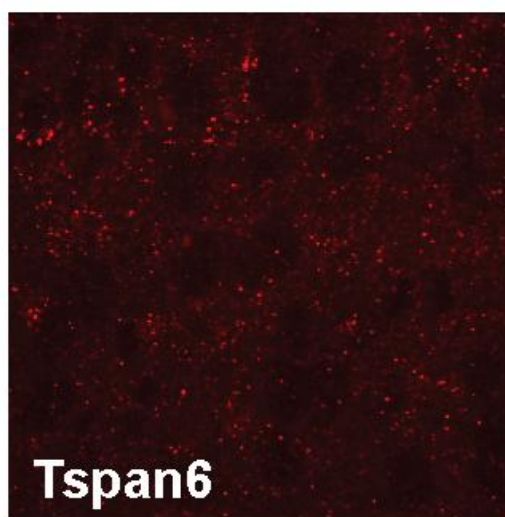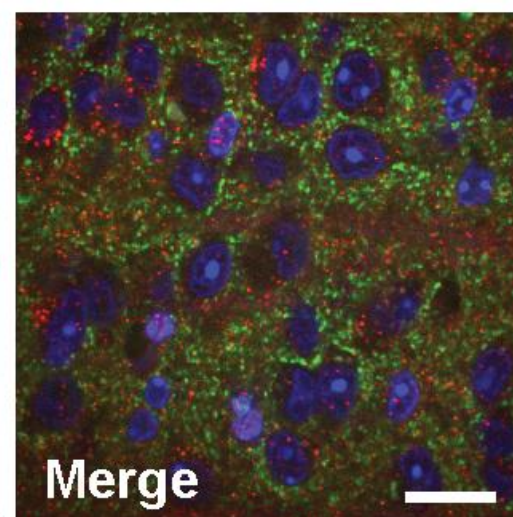

Supplement: Additional file 6: Figure S6. — Tspan6 immunoreactivity was present both in regions of high glutamatergic neuronal content (upper panels: GAD67, dentate gyrus) and high GABAergic neuronal content (lower panel: vGlut1, thalamic reticular nucleus). Scale bars = 18 μm. (PDF 119 kb) [file 13024_2017_165_MOESM6_ESM.pdf]

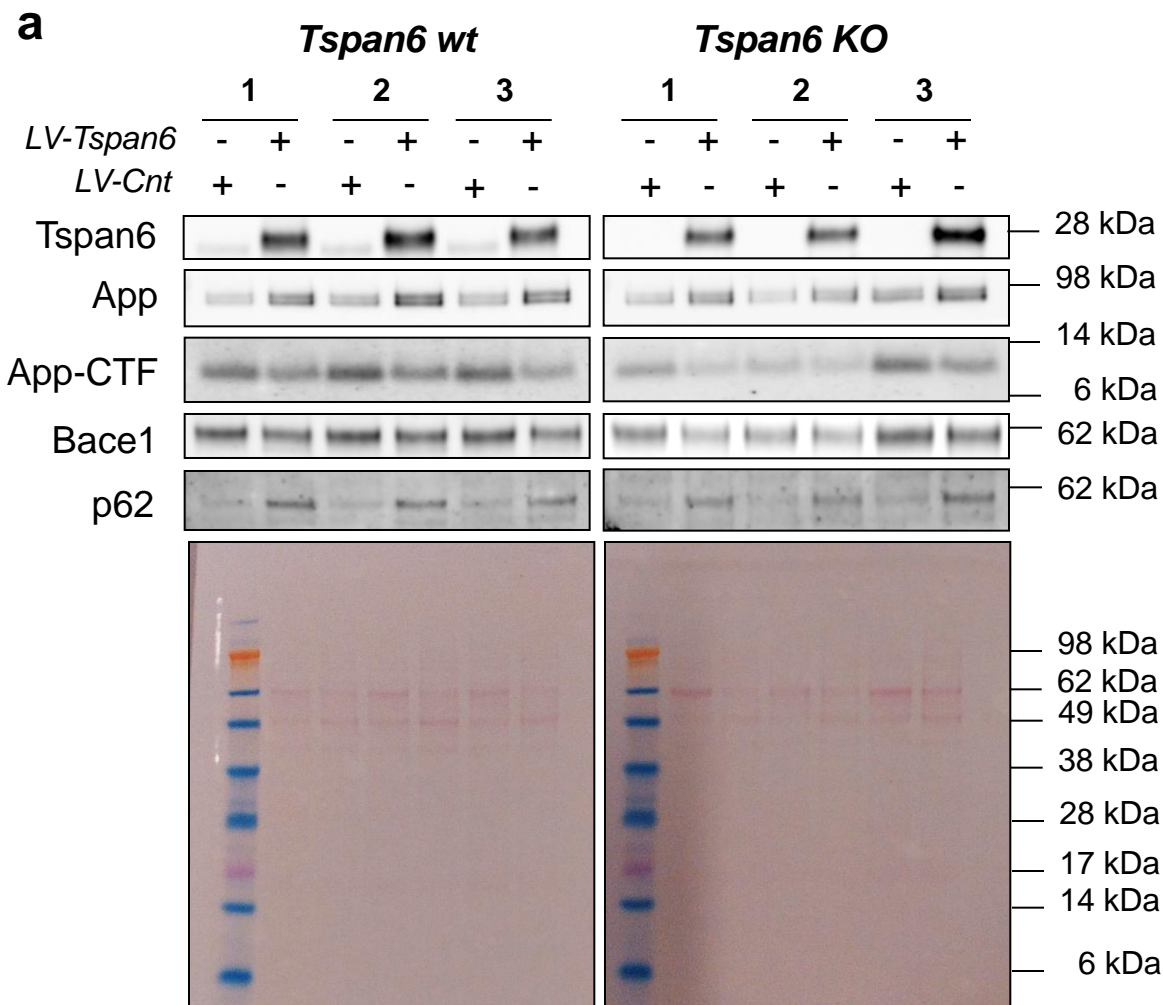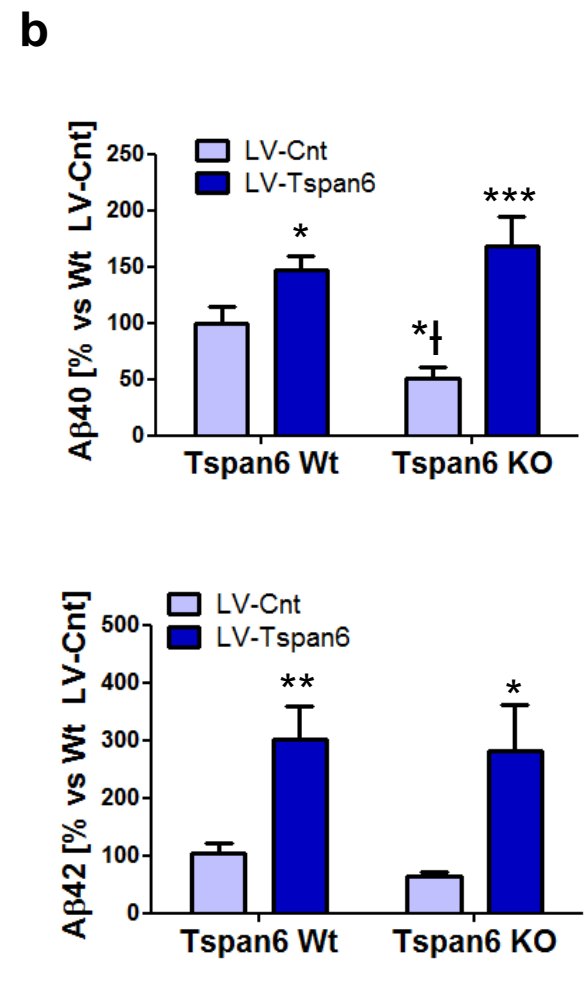

Supplement: Additional file 7: Figure S7. — a Western blots of lysates of Tspan6 wt (Tspan6 +/+ or Tspan6 Y/+) and Tspan6 KO (Tspan6 -/- or Tspan6 Y/-) primary neuronal cultures from different E14.5 embryos transduced with an empty lentiviral vector (LV-cnt) or a lentiviral vector overexpressing Tspan6 (LV-Tspan6). Overexpression of Tspan6 increases the levels of App and the autophagosomal marker p62, indicating a deficit in the autophagic pathway, but not of App-CTF and Bace1. Ponceau staining shows similar loading per well. b Increased levels of Aβ40 (upper panel) and Aβ42 (lower panel) determined by ELISA in the 12 h conditioned medium of Tspan6 wt (Tspan6 +/+ or Tspan6 Y/+) and Tspan6 KO (Tspan6 -/- or Tspan6 Y/-) primary cortical neurons transduced with LV-Tspan6 (n = 12 different E14.5 embryos for wt, n = 9 different E14.5 embryos for KO) compared to neurons transduced with LV-cnt (n = 13 different E14.5 embryos for wt, n = 10 different E14.5 embryos for KO). Statistical significance was determined by t test (*p <0.05, **p <0.01 and ***p <0.001 for comparison between LV-cnt and LV-Tspan6 conditions, *Ɨp <0.05 for comparison between Tspan6 wt and Tspan6 KO). (PDF 140 kb) [file 13024_2017_165_MOESM7_ESM.pdf]

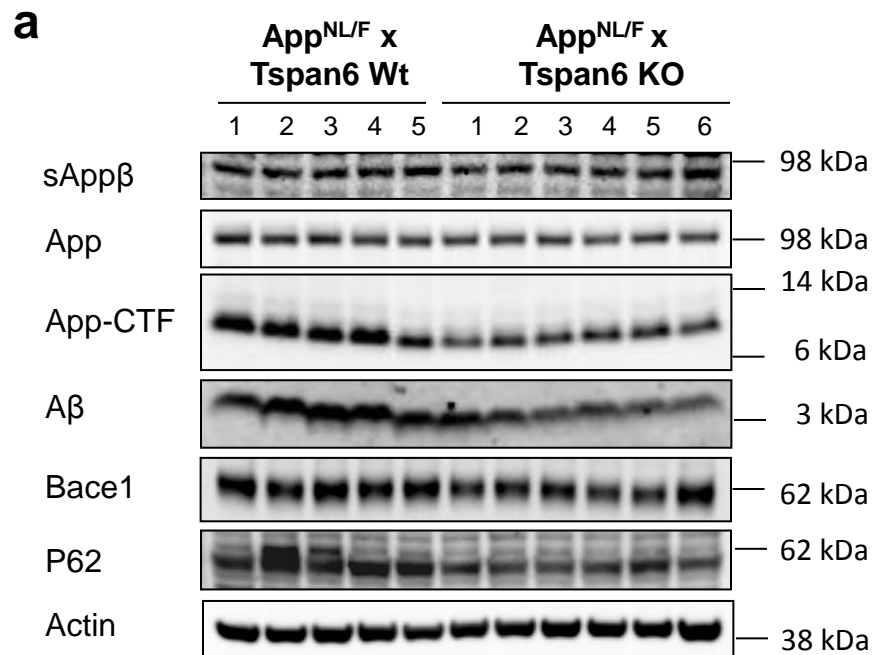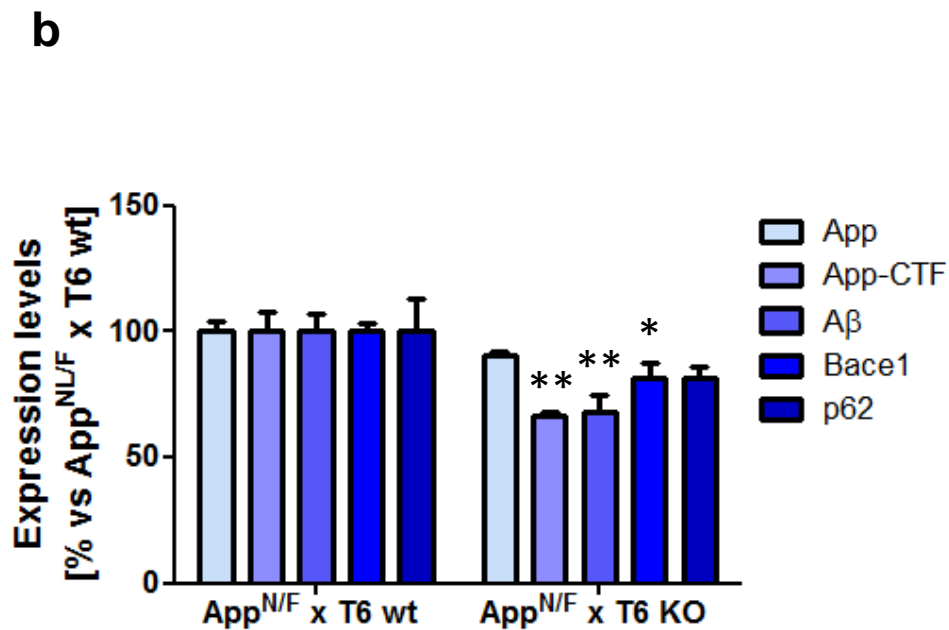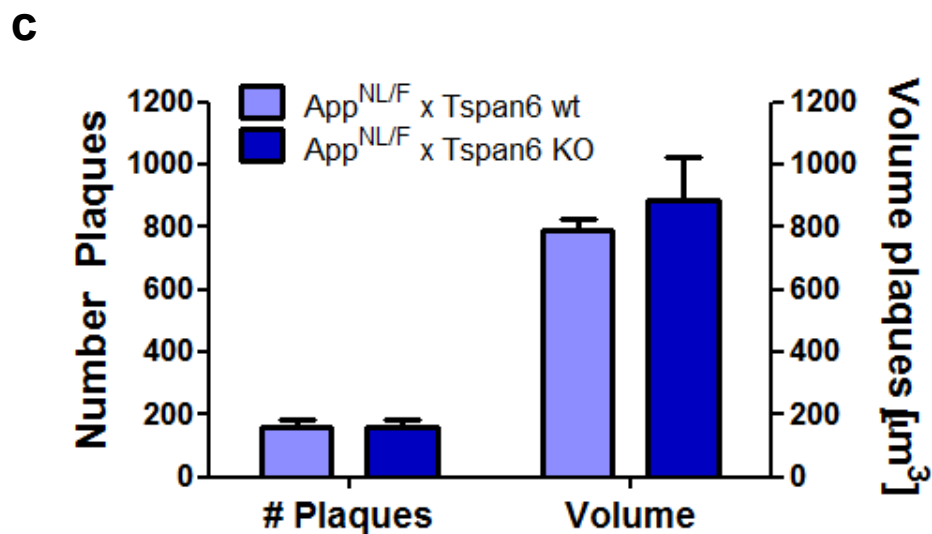

Supplement: Additional file 8: Figure S8. — a Western blot analysis of lysates from the cerebral cortex of 12 months old AppNL/F x Tspan6 wt (n = 5) and AppNL/F x Tspan6 KO (n = 6) mice. The membranes were blotted with antibodies against sAppβ, App, Aβ peptide, Bace1, p62 and actin. b Plot showing the quantification of the bands on the western blot of panel a. The levels of App-CTF, Bace1, Aβ and p62 are decreased in AppNL/F x Tspan6 KO mice (n = 6) compared to AppNL/F x Tspan6 wt mice (n = 5). c Thioflavin staining of cleared brains was performed from 1 year old AppNL/F x Tspan6 wt (n = 5) and AppNL/F x Tspan6 KO mice (n = 5) mice to visualize and quantify the 3D content of amyloid plaques. No differences in the number or the volume occupied by amyloid plaques was detected in AppNL/F x Tspan6 KO mice versus AppNL/F x Tspan6 wt mice. Statistical significance was determined by t-test (*p <0.05, **p <0.01). (PDF 168 kb) [file 13024_2017_165_MOESM8_ESM.pdf]

**a**

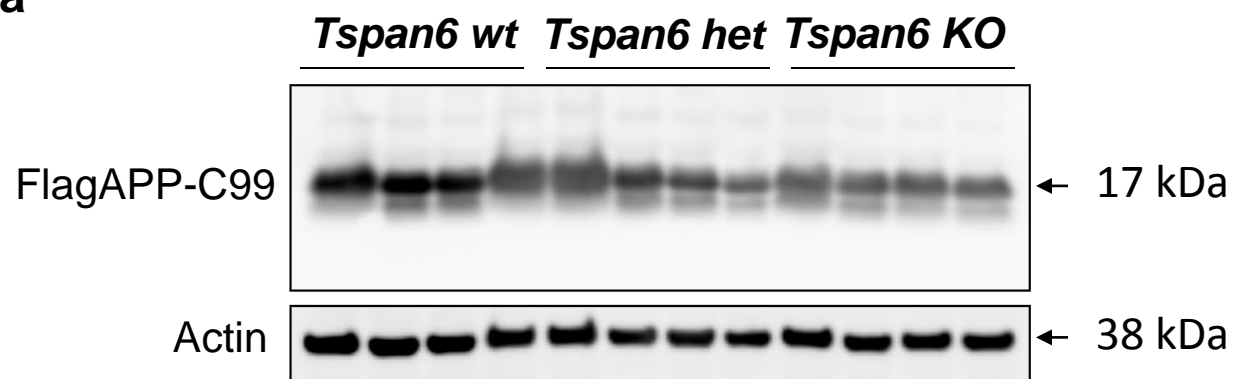

**b**

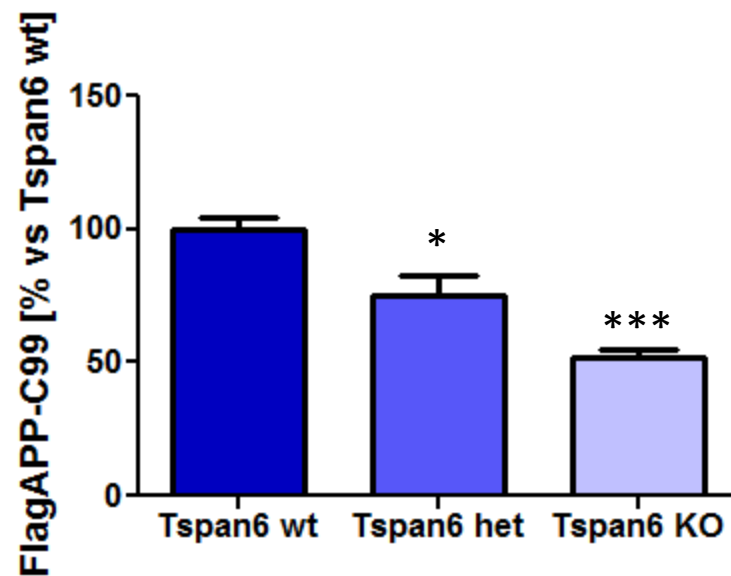

Supplement: Additional file 9: Figure S9. — a 6 DIV Tspan6 wt (Tspan6 +/+ or Tspan6 Y/+), Tspan6 het (Tspan6 -/+) and Tspan6 KO (Tspan6 -/- or Tspan6 Y/-) primary neuronal cultures were transduced with an adenoviral vector expressing the human APP-C99 triple tagged with a Flag peptide (AV-3xFlagAPP-C99) at a MOI of 100. After 48 h, 4 independent cultures of each condition were lysed and analyzed by western blot using a monoclonal antibody against Flag in order to detect the FlagAPP-C99 expression; Actin is used as a loading control. b Quantification of bands shown in panel a. Tspan6 KO neurons accumulate less FlagAPP-C99 after transduction in comparison to Tspan6 wt neurons. Statistical significance was determined by t-test (*p <0.05, ***p <0.001). (PDF 106 kb) [file 13024_2017_165_MOESM9_ESM.pdf]
